# 3D-Printed Oral Dosage Forms: Mechanical Properties, Computational Approaches and Applications

**DOI:** 10.3390/pharmaceutics13091401

**Published:** 2021-09-03

**Authors:** Danae Karalia, Angeliki Siamidi, Vangelis Karalis, Marilena Vlachou

**Affiliations:** Section of Pharmaceutical Technology, Department of Pharmacy, School of Health Sciences, National and Kapodistrian University of Athens, 15784 Athens, Greece; dankaralia@gmail.com (D.K.); asiamidi@pharm.uoa.gr (A.S.); vkaralis@pharm.uoa.gr (V.K.)

**Keywords:** 3D-printing, tablets, oral dosage forms, additive manufacturing, hardness, friability, mechanical properties, tensile strength, breaking force

## Abstract

The aim of this review is to present the factors influencing the mechanical properties of 3D-printed oral dosage forms. It also explores how it is possible to use specific excipients and printing parameters to maintain the structural integrity of printed drug products while meeting the needs of patients. Three-dimensional (3D) printing is an emerging manufacturing technology that is gaining acceptance in the pharmaceutical industry to overcome traditional mass production and move toward personalized pharmacotherapy. After continuous research over the last thirty years, 3D printing now offers numerous opportunities to personalize oral dosage forms in terms of size, shape, release profile, or dose modification. However, there is still a long way to go before 3D printing is integrated into clinical practice. 3D printing techniques follow a different process than traditional oral dosage from manufacturing methods. Currently, there are no specific guidelines for the hardness and friability of 3D printed solid oral dosage forms. Therefore, new regulatory frameworks for 3D-printed oral dosage forms should be established to ensure that they meet all appropriate quality standards. The evaluation of mechanical properties of solid dosage forms is an integral part of quality control, as tablets must withstand mechanical stresses during manufacturing processes, transportation, and drug distribution as well as rough handling by the end user. Until now, this has been achieved through extensive pre- and post-processing testing, which is often time-consuming. However, computational methods combined with 3D printing technology can open up a new avenue for the design and construction of 3D tablets, enabling the fabrication of structures with complex microstructures and desired mechanical properties. In this context, the emerging role of computational methods and artificial intelligence techniques is highlighted.

## 1. Introduction

**Figure 1:**
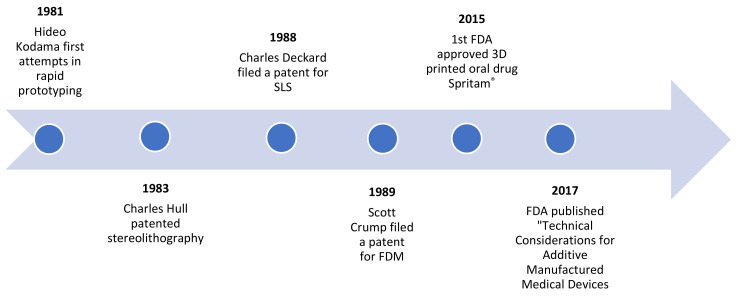
Historic timeline of 3D-printing.

Additive manufacturing or 3D printing is the construction of a three-dimensional object from a computer-aided design (CAD), adding material layer by layer [1]. The history of 3D printing is relatively recent, dating back to the 1980s. Significant technological developments have taken place over the years (Figure 1), culminating in the development of Spritam^®^, the first commercial 3D-printed oral drug to be approved by the FDA in 2015 [2]. Two years later, the FDA published “Technical Considerations for Additive Manufactured Medical Devices”, a guide for industry and Food and Drug Administration staff, and progress still continues in this promising area [3].

Undoubtedly, 3D-printing has been the focus of interest in the medical and pharmaceutical field for the last decades. For example, 3D-printing has been used in anti-cancer therapy, for the production of stimuli responsive hydrogels, nanogels, and drug-loaded implants with great flexibility and a wide variety of shapes that allow dose customization and targeted treatment with minimum side effects [4]. Another example is the application of 3D-printing in topical treatment devices. More specifically, stereolithography has been used to print a nose-shaped mask loaded with salicylic acid to treat acne. 3D-printing offers customized modeling of the mask, creating a unique geometry that perfectly matches patient’s face. Thus, this study opens a new perspective for the design of personalized devices to deal with certain pathologies [5].

Although there have been great advances in the various routes of drug administration, the oral route is still considered the most convenient and is preferred by a large number of consumers worldwide [6]. It is reported that the oral drug delivery market is expected to reach $150,063 billion by 2024 from $100,050 billion in 2018 [7]. However, dose adjustment is a common need that remains a problem as it is not addressed accurately. Tablets are divided manually using hands, knives, or tablet splitters, which leads to uneven weight distribution after division [8] and drug release problems (e.g., premature drug release, breakage of coating system, etc.) [9,10]. 3D printing, on the other hand, can effectively solve these problems by moving away from the “one-size-fits-all” approach and mass production toward personalized pharmacotherapy.

Five main techniques have been used to produce pharmaceutical dosage forms: binder jet printing, fused deposition modeling (FDM), semi-solid extrusion (SSE), selective laser sintering (SLS), and stereolithography [11]. Unlike conventional manufacturing processes, which can be time-consuming, labor-intensive, and dose inflexible due to high-volume production, 3D printing offers on-demand manufacturing at the point of care with low-cost equipment and one-step processes [12]. Dosage can be easily adapted to the specific needs of patients (pediatric or geriatric populations, patients with dysphagia, etc.) by physically altering the dimensions, geometry, or fill level of the tablet. In addition, 3D-printed tablets (printlets) are suitable for formulation development because they are inexpensive, easy to manufacture, and offer the possibility of identifying suitable active ingredients with minimal effort as early as possible in the drug development process [13]. It is also speculated that in the near future, pharmacists or clinicians could design a customized printlet with a specific dose or drug for each patient, improving treatment while reducing the risk of adverse effects [14].

However, there is still a long way to go before 3D printing is integrated into clinical practice. To date, there is no technology that can be used for large-scale production, with the exception of screen 3D printing, which was recently proposed by Moldenhauer et al. [15]. 3D screen printing is based on the transfer of API-containing printing paste through specific openings of the printing screen onto a given substrate. Unlike “conventional” 3DP technologies, which are limited by the number of print heads that build one tablet unit at a time, 3D screen printing allows thousands of units to be built simultaneously by one screen. In addition, current 3D printers are not designed for use within a good manufacturing practice (GMP) framework and lack clinical history and experience compared to more established oral dosage forms and manufacturing tools [16,17]. Nevertheless, important steps are being taken in this direction. Merck, for example, recently announced a collaboration on the 3D printing of tablets with the initial goal of GMP-compliant development and production of tablet formulations for clinical trials and, later, commercial manufacturing services [18].

**Figure 2:**
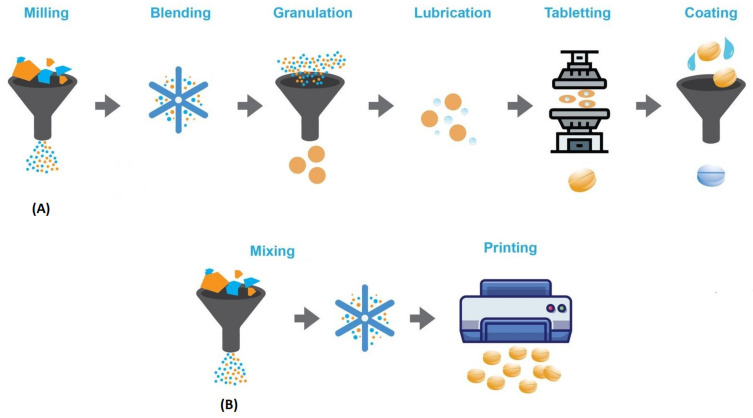
Traditional tablet manufacturing process (**A**) vs. 3D-printing manufacturing process (**B**).

Another important issue to be addressed is how to ensure quality control of 3D-printed dosage forms. To date, there is no regulatory framework for 3D-printed oral medicines, so these products should follow guidelines and meet the same quality standards as conventional dosage forms. However, this is not always possible as 3D printing techniques follow a different process than conventional oral dosage from manufacturing processes (mixing, blending, milling, and finally compression into tablets), as depicted in Figure 2. For example, in terms of mechanical strength, tablet hardness is controlled by compressive forces, whereas in additive manufacturing, different parameters determine this property [19]. Moreover, tablet hardness and friability requirements may vary between different 3D printing techniques. For example, in brittleness testing, tablets should have a weight loss of less than 1%, which is probably too stringent for tablets from binder-jet printing, which usually have low mechanical strength [13]. It is a fact that conventional mass production requires several intermediate steps (coating, packaging, distribution, etc.) before the drug product reaches the hands of the consumer intact. Therefore, it is a challenge to obtain sufficient tablet hardness and friability while maintaining the desired release properties. 3D printing, on the other hand, being a single-step process and small-scale production in on-site clinics or pharmacies, could eliminate many intermediate steps and would significantly change quality standards.

In conclusion, although the integration of 3D printed oral medicines into clinical practice is still premature, progress is being made every day and additive manufacturing will soon reach the peak of its enormous potential in the pharmaceutical field.

## 2. Physical and Printing Parameters That Affect the Tablet’s Mechanical Strength

### 2.1. Hardness

Hardness or breaking force or crush strength are very common terms in the pharmaceutical literature to describe the mechanical integrity of tablets and the force required to cause them to break in a given plane. Hardness is a very important parameter that should be monitored during product development and quality control. Tablets must be able to withstand mechanical stress during manufacturing processes, transportation, and drug distribution as well as rough handling by the end user [20]. In addition, tablet hardness is closely related to disintegration and drug release rates and is therefore particularly important for drugs that have actual or potential bioavailability problems or that are sensitive to changes in dissolution-release profiles as a function of the applied compressive force [21].

Historically, the strength of a tablet was determined by breaking it between the second and third fingers, with the thumb serving as the fulcrum. If there was a “sharp” snap, the tablet was considered sufficiently firm. Later, however, tablet hardness was defined as the force required to break a tablet in a diametrical compression test. Several hardness testers have been developed during the years such as the Monsanto, Strong-Cobb, and Pfizer hardness testers [21], however, they are not widely used today. They have been replaced by modern hardness testers, which are easy to use and provide more precise measurement results.

Most of these devices can measure thickness, diameter/length, and hardness simultaneously. There is an extended measurement range (10–500 N) and the test results are displayed on LED screens in the unit of choice (newton, kilopond or in Strong Cobb). In addition, the measurement speed can be set by the operator to multiple tests per minute and the instrument is automatically calibrated to provide accurate results [22]. Modern hardness testers are usually calibrated in kiloponds or newtons. The relationship between these units of force is: 1 kilopond (kp) = 1 kg-force (kgf) = 9.80 N. Some devices also report results in Strong Cobb units (SCU). However, the conversion between SCU and N or kp should be used with caution because SCU is derived from a hydraulic device and is a pressure [20].

There are several parameters to be considered in hardness testing such as the orientation of the tablet in the test apparatus. To ensure the comparability of results, it is best to settle on a standard orientation, either across the diameter or parallel to the longest axis [20]. For sufficient statistical accuracy, at least six tablets should be tested and the average breaking force should be within the suggested range of 3–7 kg to be considered satisfactory [23]. However, this range varies depending on the type of formulation. For example, according to the FDA guideline for industry, the hardness for chewable tablets should be kept low (12 kp). However, a higher hardness value (e.g., 12 kp) may be considered if warranted [24]. Similarly, orodispersible tablets should have a lower hardness than conventional oral tablets, etc.

Finally, it is important to note that there are differences in hardness between compressed and printed tablets. In general, 3D-printed oral dosage forms are believed to have poorer mechanical strength due to the highly porous structures, however, several studies demonstrate that 3D-printed tablets have hardness and friability values comparable to other standard pharmaceutical products [25]. In a conventional tableting press, compressive forces are used to control the hardness of a tablet. In 3D printing, however, the compressive force is not part of the process, and therefore tablet hardness must be controlled by other means such as the composition of the formulation, the drying process, the proportion of filling, the laser scanning speed, and other printing parameters, which are explained in detail below.

### 2.2. Tensile Strength

Hardness, as mentioned earlier, is the most common term for the strength of tablets. However, various factors such as inaccuracies in instrumental scale readings, zero point errors, variations in the method of load application, physical dimensions, and shape of the tablet can lead to variations in breaking strength and hence unreliable results. Although hardness is a convenient and useful parameter for in-process control and quality assurance, it is an empirical property [26]. Tensile strength, on the other hand, provides a more fundamental measure of the mechanical strength of the compressed material as it takes into account the tablet geometry. For round tablets with a flat face, the following equation is used to relate the breaking force to the tensile strength:

(1) $$\sigma_{d}=\frac{2F}{\pi \;D\cdot \;t}$$

where σ_d_ is the tensile strength (MPa); F is the breaking force (N); D is the tablet diameter (m); and t is the tablet thickness (m).

For curved face geometry, however, a more complex equation has been developed to calculate tensile strength:

(2) $$\sigma_{d}=\frac{10\;F}{{\pi \;D}^{2}}\;(2.84\;\frac{t}{D}-0.126\;\frac{t}{w}+3.15\;\frac{W}{D}+0.01{)}^{-1}$$

where W is the height of the cylindrical section of the tablet (m) and R the radius of curvature. This equation gives the tensile strength of material as a function of normalized terms (t/D, W/D, and t/D), which describe the shape and is routinely used in industrial R&D and manufacturing [27].

Increasingly, however, the pharmaceutical industry is developing more complex tablet shapes (oval, elongated, or capsule-shaped) to improve organoleptic properties such as swallowability for patients. To date, there are no relationships to calculate the tensile strength of elongated tablets, however, Pitt et al. developed an equation to calculate the tensile strength of flat and convex sided elongated tablets [28].

For flat-faced elongated tablets, the equation is:

(3) $$\sigma_{d}=\frac{2}{3}\left(\frac{2F}{\pi \;D\cdot \;t}\right)$$

For convex-faced elongated tablets, the equation is:

(4) $$\sigma_{d}=\frac{2}{3}\;(\frac{10\;F}{\pi \;D^{2}(2.84\;\frac{t}{D}-0.126\;\frac{t}{W}+3.15\;\frac{W}{D}+0.01)})$$

Generally, a tensile strength greater than 1.7 MPa is considered sufficient and ensures that the tablet maintains its structural integrity during commercial manufacture and distribution. Tensile strengths up to 1 MPa may be sufficient for small batches where the tablets are not subjected to large mechanical loads [28].

### 2.3. Friability

Tablet hardness is not an absolute indicator of mechanical strength, since some formulations, when compressed into very hard tablets, tend to “cap” or lose their crown portions when abraded. Such tablets tend to pulverize, splinter, and break, so it is very likely that they will lose their structural integrity during packaging, transportation, or handling by the end user. Therefore, friability is used as another measure of tablet strength [21].

Friability is defined as the percentage weight loss of powder on the surface of the tablets after tumbling. In the friability test, the tablets are susceptible to abrasion, so we can test the tablet strength under different force application. The friability apparatus consists of a rotating drum and exposes a number of tablets to the combined effects of abrasion and impact. For tablets with a unit weight of more than 650 mg, a sample of whole tablets is taken, corresponding to 6.5 g if possible. For tablets weighing more than 650 mg each, a sample of 10 whole tablets shall be taken. The sample shall be dusted and accurately weighed before testing. The tablets are then placed in the drum, which rotates 100 times, and are then removed, dusted, and reweighed.

The friability is calculated by the following equation:

(5) $$F=100\;\times \;(1-\frac{\mathrm{Wo}}{W})$$

where W_o_ is the initial weight of tablets (before tumbling) and W is the final weight of tablets (after tumbling).

As a rule, the test is performed once. A friability of 1.0% is considered acceptable for most products, but effervescent and chewable tablets may have different specifications. In the case of hydro tablets, a suitable humidity-controlled environment is required for the test [20].

### 2.4. Infill Percentage

Infill percentage or infill density is a parameter that determines the amount of material that occupies the inner portion of a part. In other words, it controls the percentage of printed area within the walls and top and bottom layers of the design [29]. Most 3D printers provide a range for the fill percentage between 0 and 100, with 0% making a part hollow and 100% making it completely solid.

Fill density should be carefully selected when manufacturing drugs using 3D printing, as it can greatly affect important properties of the final formulation such as weight, mechanical strength, and drug release. This has been supported by numerous studies such as by Zhang et al., who conducted design of experiments (DoE) and optimization studies and found that the fill fraction plays a significant role in tablet hardness [30]. It can be easily concluded that harder tablets are produced with increasing filling fraction. Furthermore, Thakkar et al. demonstrated that fill density can be used as an effective tool to control drug release based on patient needs and drug properties without changing the formulation composition. They describe that a 20% fill has openings in the structure that allow gastric fluids to interact with the tablet core, resulting in faster drug release, as opposed to an 80% fill that has smaller gaps and therefore allows less interaction with gastric fluids, resulting in more sustained release [29]. Goyanes et al. also support these findings as they produced fluorescein tablets with six different fill percentages (0%, 10%, 25%, 50%, 90%, 100%) and found that a higher fill percentage resulted in tablets with high mechanical strength and a prolonged release pattern [31]. However, although it is recommended to choose a high fill percentage (50%) for sustained drug release, this does not always lead to the desired result. For example, in gastrofloating tablets, relatively low fill percentages (15–30%) are preferred as they offer prolonged flotation due to lower density and higher air content [32]. Thus, if the filling percentage is chosen wisely, it can be a valuable tool in the additive manufacturing of drugs.

## 3. 3D-Printing Techniques

Various technologies have been developed for the construction of 3D printed tablets. Despite individual differences, the process involves three basic steps that are common to all techniques: (a) the creation of a computer-aided design file; (b) the conversion of the file CAD into a rapid prototyping stereolithography (.*stl*) file that describes the surface geometry of the 3D object; and (c) its conversion into a machine-specific code (.*gcode*) that is recognized by the 3D printing machine and produces the final object [33].

Some of the most common 3D printing techniques used in the pharmaceutical industry for the production of solid oral dosage forms are: (1) binder jetting; (2) fused deposition modeling; (3) semi-solid extrusion; (4) selective laser sintering; and (5) stereolithography.

### 3.1. Binder Jetting

Binder jetting (BJ) is a 3D printing technique in which a liquid binder solution is precisely applied to a powder substrate using a printer nozzle. The moistened powder particles are then fused together, solidifying the layer. The first layer is printed onto the build platform, then the plunger lowers to the thickness of the following layer and subsequent layers are printed and bonded together. The process is repeated several times until the 3D object is produced [34].

Based on these principles, Aprecia Pharmaceuticals developed the ZipDose technology that led to the production of Spritam^®^, the first FDA-approved 3D-printed tablet [Aprecia Pharmaceuticals site]. There is no doubt that BJ offers important advantages for the production of pharmaceutical dosage forms. The molding process takes place at room temperature and atmosphere, avoiding oxidation and thermal degradation of active ingredients [35]. Moreover, it is applicable to a wide range of materials and produces highly porous tablets with high drug loadings. Therefore, it is most suitable for the preparation of immediate release, fast dissolving, or orodispersible dosage forms. However, one of the major drawbacks is that it is a multi-step process requiring post-processing steps such as drying [36]. Moreover, it is particularly challenging to produce tablets with adequate mechanical properties due to high porosity. It is supported that excipient, and especially binders, play a significant role in the physical properties of tablets. Fillers with high water solubility, humectants with high water content and binders with high viscosity in solution can increase the hardness and binding strength of tablets, and consequently prolong their disintegration time [37].

### 3.2. Fused Deposition Modeling

In fused deposition modeling (FDM), drug-loaded thermoplastic polymer filaments are extruded through the print head at a specific temperature in specific directions. The molten filament is then deposited onto the build plate and solidifies in successive layers to form the object [38]. Initially, the application of this technology was established for non-pharmaceutical purposes, mainly in aerospace, architecture, and automotive industries [39] due to the lack of pharmaceutical grade polymers. However, extensive testing has been carried out over the years and today, there are a variety of polymers that can be used as matrices for drugs in FDM 3D printing. Ethyl cellulose (EC), hydroxypropyl cellulose (HPC), hydroxypropyl methyl cellulose (HPMC), hydroxypropyl methyl cellulose acetate succinate (HPMCAS), ethylated acrylate copolymer (Eudragit^®^ RL and RS), polyethylene glycol (PEG), polyethylene oxide (PEO), polylactic acid (PLA), polyvinyl alcohol (PVA), and polyvinyl pyrrolidone (PVP) are the most commonly used polymers according to the current literature.

As mentioned earlier, the use of FDM in the pharmaceutical field requires the addition of an active ingredient into the filament. This can be done either by diffusion or hot melt extrusion (HME). In diffusion, commercially available drug-free filaments are soaked with an organic solution saturated with API for more than 24 h. The filaments are then dried and ready for use. This method has been used by several researchers [31,40], but in a very low drug loading (2% *w*/*w*) and is therefore only applicable to drugs that show a therapeutic effect at low doses. There is a recent study that reports a drug loading close to 3% *w*/*w* with passive diffusion in commercially available filaments. This was achieved by developing a prediction model based on Hansen solubility theory that enables the selection of the optimal combination of drug, solvent, and filament to enhance diffusion and consequently leads to higher drug loading [41]. HME, on the other hand, involves homogeneous mixing of materials (i.e., polymer, drug, and additives such as plasticizers) and extrusion at elevated temperature to produce the polymeric filaments [42]. The printing process is usually carried out at temperatures between 150–230^O^C, above the glass transition temperature (Tg) of the polymeric materials to achieve intermixing of the compounds at the molecular level. Despite the fact that excellent drug loading is achieved (60% *w*/*w*), a major limitation of this technique is the thermal degradation of thermosensitive drugs. One way to overcome this is to select miscible polymers with low glass transition temperature, leading to a decrease in the overall melting temperature of the powder mixture, thus reducing the risk of API degradation due to high temperatures [43].

In contrast to BJ, which produces highly porous tablets, FDM is mainly preferred for the production of modified release dosage forms because it produces tablets with complex geometries, excellent hardness, and low friability. In addition to the excipients, temperature also affects the mechanical properties. A high temperature (above the Tg of the polymers) during the printing process allows molecular diffusion and consequently strong interfacial adhesion between the adjacent layers of the molten material. However, too high a temperature could create air pockets between the layers and induce porosity, which reduces the mechanical strength of the final formulation [44].

### 3.3. Semi-Solid Extrusion

Semi-solid extrusion (SSE) is a 3D printing technique in which material in semi-solid or semi-molten form is extruded from a syringe-like system in successive layers to form a three-dimensional object. Unlike FDM, which uses solid filaments, SSE prepares the starting material by mixing the ideal ratio of active substances with solvents to form a gel or paste [45]. Moreover, low temperatures are used during the process, therefore, it is suitable for thermolabile active ingredients. It has been applied to the preparation of various dosage forms including immediate release tablets, orodispersible tablets, pediatric gum formulations, controlled release tablets, gastrofloating tablets, and solid lipid tablets. It is a versatile and simple technique as the drug can be mixed directly with excipients and filled into a syringe or cartridge. Therefore, it can potentially be used in clinical practice, in local pharmacies or clinics, to produce customized and personalized formulations [46,47]. However, SSE has some disadvantages that need to be considered. The excipients should be carefully selected to be miscible and have the appropriate viscosity and rheological properties to facilitate the printing process. For example, low viscosity results in excessive material flow and high viscosity results in inadequate material flow. In addition, a post-processing step of drying or cooling is often required. However, the physical state of the starting material can affect the drying process, potentially leading to shrinkage or deformation of the product or collapse of the object in case of insufficient hardness [36]. Finally, unlike FDM, SSE prints at low resolution, mainly because the die heads of these printers need to be wide enough to accommodate a highly viscous material, compared to the extrusion orifices of other printers [48].

### 3.4. Selective Laser Sintering

Selective laser sintering (SLS) is a 3D printing process that uses a powder bed as the starting material, similar to BJ. However, the main difference between these two techniques is that SLS uses a laser beam to heat and fuse the powder particles together, rather than using a liquid binder solution. During the printing process, the first layer of powder is spread evenly on the build platform and then the laser draws a specific pattern on the surface of the powder bed. Once the first layer is completed, a roller spreads a new layer of powder on top of the previous one, and as the process continues, a 3D object is gradually formed [49]. SLS is a one-step process that does not require prior preparation of filaments as in FDM or post-processing steps such as drying in BJ and SSE. It is also a solvent-free process that prints at high resolution due to its high laser precision. However, it is mostly used in non-medical manufacturing industries for the production of plastic, ceramic, or metal objects where high temperatures and high energy lasers are required for sintering [50,51]. In the pharmaceutical industry, SLS has so far had limited application due to the possible drug degradation caused by the high energy input of the laser [52]. Nevertheless, there are several studies on SLS-printed oral dosage forms, mainly orodispersible and immediate-release tablets. This could be because SLS offers the advantage of controllable porosity and reproducibility by modifying the printing parameters such as laser scanning speed. The laser scanning speed is effectively the speed at which the laser beam travels when drawing the 3D pattern and has a significant effect on the mechanical properties of the final formulation. When the speed is low, the contact time between the powder bed and the laser beam is increased, resulting in harder and denser tablets being produced. On the other hand, when the scanning speed is high, less energy is transferred to the powder, resulting in less sintering and consequently more porous and brittle structures [53].

### 3.5. Stereolithography

Stereolithography (SLA) is an additive manufacturing process in which the object is created by selectively curing a polymer resin layer by layer with an ultraviolet (UV) laser beam. The materials used in SLA are photosensitive polymers that are in liquid form. In some cases, post-curing with a UV oven can be used to increase the mechanical strength of the object [54]. In the medical field, stereolithographic 3D printing is mainly used in tissue engineering [55,56,57,58] and in the fabrication of implantable devices [59,60]. However, applications in the pharmaceutical field are still limited.

One of the major advantages of this technique is the high printing resolution, which is superior to other 3D printing techniques. It also minimizes local heating during the process, making it suitable for the fabrication of oral dosage forms containing thermolabile drugs. This was experimentally demonstrated by Wang et al. who successfully used SLA to print tablets containing 4-ASA, a known thermosensitive drug. It was found that SLA reduced thermal drug degradation compared to FDM [61]. However, the main reason holding back the application of SLA in pharmaceuticals is the limited number of photocrosslinkable polymers that are safe for pharmaceutical use [62]. There could be potential hazards from the use of resins such as carcinogenicity, which need to be identified in the future. In addition, due to the photosensitivity of the starting material, it is likely that the drug formulations could have issues with long-term stability [6]. Finally, there is the possibility of incompatibility between the photopolymers and the APIs. For example, Xu et al. used SLA printing to prepare an antihypertensive polypill with four different APIs. However, an unexpected reaction occurred between the photopolymers and one of the APIs, indicating that compatibility issues should be considered prior to the printing process [63].

## 4. Methods

Several approaches have been taken to ensure a high-quality literature review dissertation of 3D printed oral pharmaceutical formulations, focusing on their mechanical properties. Two main databases—Science Direct and Google Scholar—were used for an initial comprehensive and a second in-depth search of the topic. The main keywords used in the search were: 3D printed tablets, 3D printed oral dosage forms, additive manufacturing, hardness, friability, mechanical properties, tensile strength, and breaking strength. Significantly, the search “hardness AND 3D-printed oral dosage forms” with an adjusted time frame of 2014 to 2021 yielded 1030 articles in Google Scholar and 214 articles in Science Direct. Only research articles were selected from this set and duplicates were removed. In addition, studies on non-oral dosage forms or studies on oral films were excluded. Studies without sufficient data on hardness and friability were also omitted and the final number of articles analyzed in detail in the Results and Discussion section was 34. Finally, the United States Pharmacopeia, Food and Drug Administration (FDA), and European Medicines Agency (EMA) websites were also used to obtain reliable information on the guidelines on hardness and friability of oral dosage forms and the protocols that must be followed in the industry today.

## 5. Results

After extensive study of the current literature, 34 research articles were carefully selected and analyzed to draw some conclusions about which excipients and printing parameters affect the mechanical properties of 3D printed oral dosage forms. To achieve this, summary tables were prepared and the studies were grouped mainly by drug release mechanism. The release behavior was reported as indicated by the author(s). If the author(s) did not characterize the release rate, the following rule was applied: if the drug release was ≥ 75% within 30 min, the profile was characterized as immediate. In all other cases, the release profile was characterized as modified. At a second level, studies were classified by dosage form (e.g., pediatric formulations, orodispersible tablets, gastrofloating tablets, etc.). In addition to the active ingredient, the table also included the main excipients and the techniques used. Finally, the quantitative and qualitative test results on hardness, friability, and tensile strength were used to make the relevant comparisons and reach the conclusions.

The studies in Table 1 refer to immediate release formulations. Table 2, Table 3 and Table 4 do not relate to release mechanism but contain data from preformulation studies and provide useful information on the influence of excipients on the mechanical properties. The studies in Table 5 are arranged according to the mechanism of drug release, so we had seven main categories: modified release, controlled release, gastro-floating tablets sustained release, delayed/biphasic release, delayed/controlled release, and delayed release. Some of these categories are further subdivided based on dosage form to facilitate the process of comparing and drawing conclusions. 

### 5.1. Immediate Release

#### 5.1.1. Pediatric Formulations

**Figure 3:**
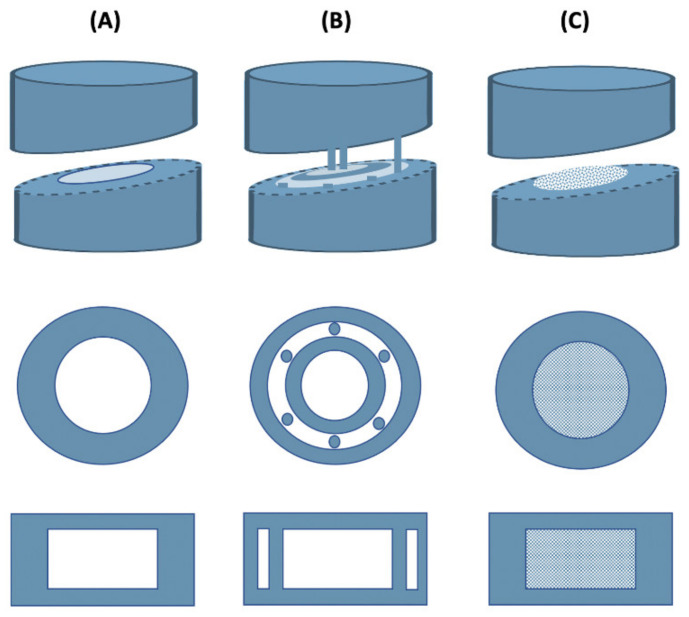
3D-printed levetiracetam pediatric formulations. (**A**) Hollow structure, (**B**) Hollow structure with internal support, (**C**) Lattice structure.

Formulation of drug dosage forms for the pediatric population remains a challenging task due to low doses required, limited swallowability, lack of adherence due to poor taste, etc. [79]. Liquid dosage forms are usually preferred in this case, but they also have potential drawbacks such as stability issues, limited control over dose absorption, or limited ability to modify drug release, which often limit their use [80]. Due to its great potential, 3D printing has contributed greatly to the production of pediatric solid dosage forms with precise dosing and child-friendly appearance and taste. For example, a group of researchers combined binder jet with color jet 3D printing to produce pediatric immediate-release preparations of levetiracetam. The drug was mixed with microcrystalline cellulose, mannitol, and sucralose to form the printing powder, while appropriate proportions of binder (PVP K30), plasticizer (glycerol), and wetting agent (polysorbate 20) were added to isopropanol to produce a suitable ink. It was found that the addition of PVP to the ink had no effect on tablet hardness, but significantly reduced tablet friability. When the PVP content in the ink was less than 0.1% (*w*/*w*), the hardness of the tablets changed little with increasing PVP content, but the friability decreased from 10.70% to 5.9%. However, too high a PVP content (more than 0.5% (*w*/*w*)) resulted in higher friability, so 0.05% PVP was chosen as the optimum percentage. In addition, preliminary tests showed that the addition of glycerol to the ink at percentages within 4% (*w*/*w*) could significantly improve the tablet formability, but a higher glycerol content (6–8% *w*/*w*) would result in very high friability values (9%). As for the tablet structure, three different models were used (lattice structure, hollow structure, and hollow structure with internal support) (Figure 3) to test their influence on the mechanical properties and drug release. The hollow structure with internal support resulted in tablets with a very low average hardness (13 N), which also failed the friability test. On the other hand, the average hardness of the tablet with hollow structure and the tablet with lattice structure were 61 and 59 N, respectively, and the tablet remained intact after the friability test. The hollow structure was chosen as the final tablet model because it showed similar mechanical properties, but faster disintegration than the lattice structure. Finally, hollow structure cartoon tablets with different strengths (1000 mg and 250 mg) and appearances (rabbit, candy, heart, bear) were designed and showed good hardness (21.05–59.21 N) and friability (2.77–5.75%) similar to the commercially available Spritam^®^ [64].

Tagami et al. worked with another antiepileptic drug, lamotrigine, to produce gummy formulations for pediatric use with an extrusion-based 3D bioprinter using a hydrogel as the ink. The hydrogel consisted of gelatin, HPMC 2208, drug, reduced starch syrup, and water. Different amounts of gelatin and HPMC were also used to investigate how they affect the mechanical properties of the printer ink and printed formulations. More specifically, gelatin hydrogel formulations were prepared without HPMC, drug, and reduced syrup as the control (Table 2) and the results showed that as the amount of gelatin increased, the hardness of the formulations also increased. The same conclusion was drawn after preparing drug formulations with HPMC, reduced syrup, and increasing amounts of gelatin, indicating that the presence of the latter greatly affects the hardness and texture of the gum. In addition, the strength of the formulation without gelatin could not be measured. Finally, the incorporation of HPMC into the gummed drug formulation increased the viscosity, but had no effect on or slightly decreased the strength, while the addition of the drug to the gummed formulation had no effect on the hardness [65].

**Table 2:** Pre-formulation test on the impact of gelatin on hardness of gummy formulations [65].

Excipients	Breaking Force (N)
Gelatin 5%	~ 1.8
Gelatin 10%	~ 5
Gelatin 15%	~ 10

Karavasili et al., on the other hand, developed chewable chocolate-based dosage forms using 3D extrusion printing. They used both a lipophilic (ibuprofen) (BCS class II) and a hydrophilic (paracetamol) (BCS class I) drug as model drugs with successful results. In addition to dark chocolate as the main excipient to mask taste and improve organoleptic properties, corn syrup (glucose syrup) was also used to facilitate drug loading. However, this affected the hardness of the final dosage form. It is reported that incorporation of the syrup into the chocolate matrix significantly reduced the hardness of the chocolate-based dosage forms, which is a preferred textural property as it makes them easier for children to chew. The final dosage forms allowed rapid and high dissolution of both drugs in simulated salivary fluid, making them readily available for absorption in the oral cavity and via the gastrointestinal tract during swallowing. This would be particularly beneficial for drugs with high permeability and low water solubility such as ibuprofen [66].

#### 5.1.2. Orodispersible Tablets

Orodispersible or orally disintegrating tablets (ODTs) can be defined as uncoated tablets intended to be placed in the mouth, where they dissolve readily within 3 min before swallowing. They offer several advantages (e.g., stability, administration without water, accurate dosing), making them particularly useful in pediatric and geriatric populations or patients with swallowing difficulties [81]. However, ODTs are characterized by low mechanical strength and high friability, so they require careful handling by the end user.

In recent years, 3D printing has become increasingly popular for the fabrication of ODTs, as shown by several recent studies. For example, a scientific group has produced orodispersible (flash) carbamazepine tablets with SSE, using cyclodextrins to increase the solubility and bioavailability of the drug [67]. Besides hydroxypropyl-β-cyclodextrin (HPβCD), HPMC F4M (binder) and croscarmellose sodium (disintegrant) were also used as excipients. Hydroxypropylmethylcellulose (HPMC) polymers are commonly used as excipients in the preparation of hydrophilic tablet matrices to control drug release. However, several types of HPMC are commercially available, which differ in their molecular size and chemical substitution (the proportion of methoxy and hydroxypropyl substituents), affecting compactability and drug release [82]. HPMC E4M (medium substitution ratio) was also tested as a potential binder in preliminary experiments, but provided wet masses that were too hard and were therefore discarded. HPMC F4M (high substitution ratio), on the other hand, allowed the preparation of homogeneous mixtures without lump formation. Croscarmellose is a cross-linked derivative of carboxymethylcellulose sodium that swells strongly on contact with water and offers very rapid disintegration [83]. Regarding the physical properties of the tablets, they exhibited a breaking force value of 25 N and friability above the 1.0% limit (2.2%), but still showed suitable physical properties for handling. Allahham et al. also worked with cyclodextrins as drug carriers but used a different 3D printing technique, selective laser sintering, to produce ondansetron-orodispersible tablets. Ondansetron was incorporated into cyclodextrin complexes to combat the bitter taste of the drug and thus improve patient compliance. Mannitol was also used as a filler due to its taste masking properties. More specifically, two different formulations were prepared, both containing 22% (*w*/*w*) ondansetron–cyclodextrin mixture (1:5 ratio) and 3% (*w*/*w*) of gold pigment, but different proportions of copovidone VA 64 and mannitol. Copovidone VA 64 is a vinylpyrrolidone-vinyl acetate copolymer that provides good printability and fast disintegration properties, and gold pigment, a dye that helps the powder mixture absorb the maximum amount of energy from the laser beam at this wavelength (445 nm), resulting in a successful sintering process. In addition, preliminary tests were conducted to evaluate different laser scanning speeds, and a laser speed of 200 mm/s was selected as optimal. The results of the hardness tests showed that the formulation with the highest mannitol content (60%) had a slightly higher hardness (18.5 N) than the formulation with 50% mannitol (14.7 N), although a reduction in mechanical properties was expected. It was also found that only a small percentage of copovidone VA 64 (15% *w*/*w*) was sufficient to maintain the 3D printed formulations and preserve the structure of the printlets, allowing the use of 82% *w*/*w* for other materials such as drugs (allowing higher drug loading) or excipients such as mannitol or cyclodextrins (for taste masking effects). Finally, it is noted that both formulations do not break during manipulation and have sufficient mechanical properties for easy handling. Even though their breaking strength values are reduced, the fact that there is no minimum breaking strength requirement for ODT formulations would make them suitable when conditioned in blister packs [68]. SLS has also been used by another group of researchers to prepare orally disintegrating paracetamol printlets [69]. Two different formulations were prepared, one using the HPMC E5 polymer and the other using the copovidone VA 64 polymer, and different laser scanning speeds were applied (100, 200, 300 mm/s) to obtain three different types of formulations for each polymer. The formulations prepared with higher laser scanning speeds were lighter, had higher porosity and lower breaking strength, which is due to the fact that increasing the speed reduces the sintering of the powder particles together and leaves more voids between each particle. This leads to a fast disintegration profile, which is optimal for ODT formulations. On the other hand, printlets printed at lower laser speeds exhibited higher density and hardness as well as a slower disintegration profile, as the powder particles did not separate as easily when in contact with the dissolution medium. However, all the formulations showed suitable properties for handling and did not break easily when manipulated. Finally, of all the printlets prepared, only the copovidone printlet prepared at a laser scanning speed of 300 mm/s met the EU and US pharmacopeia criteria for ODTs and was able to dissolve rapidly within 4 s. Similarly, Awad et al. produced paracetamol tablets using the SLS technique, although these printlets brought the unique property of being used by patients with blindness or visual impairment [70]. More specifically, all 26 alphabets were printed on the surface of the printlets in the form of Braille and lunar alphabets, which enables patients to overcome everyday problems in pharmacotherapy (e.g., identifying the drug when it is taken from the original container, taking the wrong drug, wrong dosage, difficulty in remembering the instructions, etc.). Copovidone VA 64 and gold pigment were used as the main excipients and the laser speed was set at 300 mm/s. As for the mechanical properties, a traditional hardness test was performed on printlets without pattern, printlets with braille letter A, and printlets with braille letter Q. The mechanical properties of the printlets were tested. The results showed that the addition of the patterns did not affect the mechanical properties of the printlets and all printlets had similar breaking force values (13.9–14.5 N), which were comparable to the 3D-printed ODTs discussed previously [68,69].

#### 5.1.3. Tablets

Immediate-release tablets or capsules are used when the rapid onset of action of a drug is advantageous [84]. According to the EMA reflection paper, immediate release is referred to as a dissolution of at least 75% (Q) of the active ingredient within 45 min [85]. On the other hand, the FDA guidance for solid, orally administered IR drugs that are intended to be swallowed and contain a highly soluble active ingredient states that the dissolution criterion is Q = 80% in 30 min [86]. Undoubtedly, there are numerous scientific reports on 3D printed tablets with immediate release. Conceição et al. also prepared immediate-release carbamazepine tablets with SSE using cyclodextrins as solubilizers, in addition to the orodispersible carbamazepine tablets discussed previously. HPMC F4M was used in both formulations, but in orodispersible tablets, HPMC was partially replaced by croscarmellose sodium to accelerate disintegration. IR tablets showed higher breaking strength value (35 N) than ODTs (25 N) and lower friability (1.8%) compared to ODTs (2.2%). Although the friability was higher than the accepted limit, it was found that the tablets could be easily handled by the end users. Finally, a relationship was observed between the breaking strength of the prints and the disintegration time (i.e., the most fragile tablets (orodispersible) showed a shorter disintegration time) [67]. There is also another study on paracetamol tablets with immediate drug release produced by extrusion-based 3D printing [71]. This technique allowed for very high drug loading (80% *w*/*w*) in contrast to direct compression, which has the disadvantage of relatively low drug loading. Polyvinylpyrrolidine (PVP K25) was used as a binder and croscarmellose sodium (NaCCS) as a disintegrant, producing tablets that showed disintegration in less than 60 s and release of most of the drug within 5 min. The filling fraction was 100% and the printed tablets showed high density and acceptable mechanical properties, with an average breaking strength of 78.13 N and 0.54% friability. In addition, the tensile strength of the tablets was measured and the average was 8.93 MPa, indicating high resistance to fracture and mechanical impact. Generally, a tensile strength greater than 1.7 MPa is considered sufficient and ensures that the tablet maintains its structural integrity during commercial manufacture and distribution [28]. As mentioned by the authors, the binder is essential for controlling the tablet breaking force in 3D printing, as opposed to the compression forces that control the tablet breaking force in conventional tableting presses. PVP, HPMC, and hydroalcoholic gels are some of the most common binders used in extrusion-based 3D printing. The same scientific group also experimented with tablet geometry and fabricated paracetamol mesh tablets using an extrusion-based 3D printer and PVP K25, NaCCS, and starch as excipients [72].

Dissolution studies showed that more than 70% of the active ingredient was released within the first 15 min, which can be attributed to both the tablet geometry and the inclusion of disintegrators. More specifically, it is referred to the fact that the increased surface area allows rapid water uptake by the NaCCS, leading to rapid disintegration. As for the mechanical properties, the printed tablets showed sufficient tensile strength (2.24 Mpa) and friability (0.65%), but the breaking force measurements did not reach the minimum satisfactory value. Nevertheless, the 3D-printed paracetamol tablets appeared to be quite robust and could tolerate a reasonable amount of rough handling [72]. Li et al. experimented with a different technique to produce rapid-release puerarin tablets. They used thermal extrusion, in which molten thermoplastic semi-finished products are applied through a hot nozzle at much lower temperatures than the FDM process, avoiding material degradation. In addition, no solvent was used; only PEG 4000 was chosen as a suitable carrier to achieve rapid release. Polyethylene glycols are polymers with hydrophilic properties, low toxicity, and low melting points (usually 65^o^C), which are advantageous for the preparation of solid dispersions by the melting process [87]. PEG 4000 was used in different ratios and all the tablets prepared showed good hardness (79–138 N) and friability (1%). With the increase in PEG 4000, an increase in hardness and drug release rate was observed while the friability was kept consistently low despite the changes. It was also demonstrated that PEG 4000 could improve the solubility of puerarin at a ratio of puerarin to PEG 4000 of 1:5, so this was chosen as the optimal ratio [73]. In addition, there was an interesting recent study describing the preparation of clindamycin palmitate hydrochloride 3D-printed tablets (printlets) by SLS [74]. They prepared a batch of fifteen tablets and used the Box–Behnken design to investigate how laser sintering speed, microcrystalline cellulose (MCC) concentration, and lactose monohydrate (LMH) concentration affect important properties of the final formulation such as hardness and dissolution time. The MCC and LMH concentrations were in the range of 5–10% and the laser scanning speed was 200–300 mm/s. The results of the hardness test showed that the tablet hardness varied from 7.3 N to 18.3 N and was strongly affected by the laser scan speed. By changing only the laser speed and holding all other parameters constant, it was proven that a slower speed produced mechanically firmer dosage forms because the dwell time of the laser to interact with the powder was longer. Similarly, MCC increased mechanical strength due to its binding ability. Moreover, a synergistic binding effect was observed by the combination of copovidone VA 64 and MCC. On the other hand, LMH decreased the mechanical strength of the tablets, which is probably related to the high melting point of LMH compared to copovidone VA 64. The high melting point of LMH results in it being either in an unmelted or partially melted state, which disturbs the molecular mixing of the components and thus reduces the mechanical strength of the tablets. Laser scanning speed was the most important process factor, MCC concentration had a statistically significant effect, while LMH concentration had a lesser effect on the mechanical properties of the tablets. Finally, all formulations exhibited a disintegration time of 5 min, while 79.3% to 96.8% of the drug was released in 30 min, indicating an immediate release profile. An increase in hardness was found to increase the disintegration time and consequently the time required for dissolution of the drug [74].

Hussain et al. prepared rapidly dissolving captopril tablets for the treatment of hypertensive crisis using the FDM/HME technique [75]. The main excipients used were HPC-SL (binder), PEG 6000 (plasticizer), and two different types of disintegrants: sodium starch glycolate and croscarmellose sodium. HPC is a cellulose ether in which some of the hydroxyl groups of the repeating glucose units are substituted by –OCH_2_CH(OH)CH_3_ groups with the help of propylene oxide. The degree of hydroxypropyl substitution can be changed to adjust the viscosity of the polymer. In this study, the HPC SL type was selected as a low viscosity polymer to facilitate HME and to produce a chemically stable mixture that avoids thermal degradation of the drug [88]. In addition to captopril tablets, blank tablets consisting only of HPC and PEG 6000 were also prepared to test the effect of excipients on mechanical properties (Table 3). All tablets were printed with 100% fill, exhibited zero friability, and high hardness (385 N) exceeding the upper limit of a common hardness tester. Moreover, the uniformity of hardness values among all formulations is attributed to the identical 3D structure and design of these tablets. Finally, according to the dissolution studies, sodium starch glycolate tablets showed a release of about 40% of the drug within 15 min, while croscarmellose sodium tablets released 50% of the drug in the same time. Both formulations showed almost complete release in 2 h, indicating an immediate release profile [75].

**Table 3:** The effect of excipients on the mechanical properties of blank (without drug) tablets produced with the FDM/HME technique [75].

Excipients	Breaking Force (N)	Friability (%)
HPC- SL PEG 6000	385.1	0

**Figure 4:**
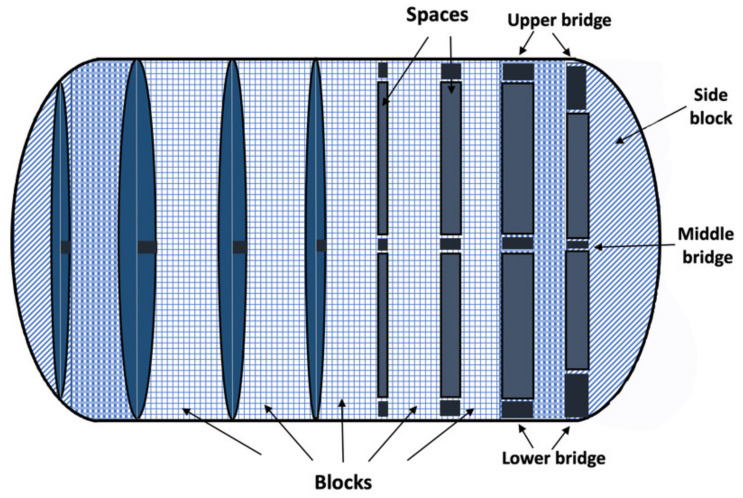
Immediate release theophylline gaplets. The novel design is based on nine repeating units (blocks) joined together by three bridges. The capsule-like general shape was maintained by using curved side units [76].

On the other hand, a group of researchers pursued a novel design approach to accelerate decay and drug release without the use of disintegrators [76]. They fabricated theophylline tablets with unique built-in gaps (gaplets) using the FDM technique. The drug-loaded filaments were fabricated by HME and consisted of theophylline, hydroxypropylcellulose (binder), and triacetin (plasticizer). The novel design (Gaplet) was an articulated system in which the tablets were produced in a capsule-like structure divided into nine blocks with certain eight spaces between them (Figure 4). The gaps varied from 0–1.2 mm (0, 0.2, 0.4, 0.6, 0.8, 1.0, and 1.2 mm) [76]. Tablets without or with smaller spacing showed slow drug release, but with larger spacing, drug release was accelerated due to less resistance of the medium flowing through the tablet structure. Furthermore, the addition of disintegrants to the formulation had no significant effect on the release profile, suggesting that gaplets are superior to a more conventional formulation approach that includes disintegrants to achieve rapid disintegration and dissolution. Finally, the “gap design” had an impact on the mechanical properties of the printed tablets, with the solid tablets (without gaps) exhibiting a fracture strength of 227 N, compared to 17 N for the 1.2 mm spaced tablets. This shows that increasing the spacing between the blocks reduces the structural strength, causing the bridges between the blocks to fail under the applied stress. However, the robustness of the tablets was not affected as indicated by the zero values of friability obtained for all designs [76].

Interestingly, Tian et al. investigated the effect of different excipients on hardness, friability, and disintegration time by producing BJ printed tablets without an active ingredient (Table 4). They found that fillers with high water solubility (e.g., D-sucrose) can improve the formability and bonding ability of the printed tablets while increasing their hardness. This is mainly because the soluble excipient is dissolved in an appropriate volume of water during wetting and then dried to crystallize a “solid bridge” between adjacent particles. PEG 4000 was tested alongside other excipients as a binder, but provided poor bonding, resulting in unacceptable hardness and friability values. Finally, the 10% ethanol wetting solution exhibited the highest solubility of d-sucrose (as it is highly soluble in water and sparingly soluble in ethanol), resulting in tablets with greater binding and hardness as well as lower friability. The greater binding also resulted in a longer disintegration time as the d-sucrose-10% ethanol formulation disintegrated within 26.4 s, while d-sucrose-30% ethanol disintegrated in 17.1 s and d-sucrose-60% ethanol disintegrated in 10.2 s [89]. Therefore, these data can be used as a reference point for the preparation of 3D printed immediate release tablets.

**Table 4:** The effect of various excipients on the mechanical properties of tablets printed with the binder jet technique [89].

Excipients	Breaking Force (N)	Friability (%)
d-sucrose, 50% ethanol	20.27	0.38
Mannitol, 50% ethanol	11.22	3.24
d-sucrose, PEG 4000, 50% ethanol	10.96	3.51
d-sucrose, 10% ethanol d-sucrose, 30% ethanol d-sucrose, 60% ethanol	36.56 28.02 18.24	0.13 0.29 0.41

The BJ technique was also used in another study in which immediate-release tablets of amitryptiline hydrochloride were prepared [77]. The innovation of this study was to achieve effective and precise drug loading, even at the microscale, by incorporating the drug into the ink rather than adding it to the powder mixture. The powder mixture consisted of lactose monohydrate, di-calcium phosphate anhydrate, and PVP K30. Dissolution studies showed that more than 80% of the drug was released within 30 min and the disintegration time of the printed tablets was less than 2 min. This shows the advantage of the BJ 3D printing process over other printing processes such as FDM, where the disintegration time of the produced tablets was at least ~5 min. The hardness (35–49 N) and friability (< 0.87%) values were found to be adequate, indicating the potential use of binder-jet 3D printing for the fabrication of IR dosage forms.

Finally, there is a very recent study applying SSE to print solid lipid tablets from emulsion gels [78]. The poorly water-soluble fenofibrate was selected as a model drug and three different lipid-based formulations were prepared and then converted into printable emulsion gels by adding methylcellulose [78]. Moreover, the addition of croscarmellose sodium to these formulations ensured rapid disintegration (15 min), which is consistent with the requirements for immediate release tablets. Finally, the 3D-printed tablets showed suitable mechanical properties for easy handling without the risk of fracture or deformation. However, a hardness test of the 3D-printed tablets could not be performed after drying, as they were softer than the compressed tablets. This was to be expected as compression forces are not part of the 3D printing process in traditional tablet manufacturing. In the case of SLS, tablet hardness can be controlled by including a binder in the formulation [78].

### 5.2. Modified Release

**Table 5:** Composition of 3D-printed solid oral dosage forms linked with technique % infill percentage, breaking force (N), friability (%), and grouped according to their modified release behavior.

Release * Behavior	Dosage Form	Technique	% Infill	API	Excipients	Breaking Force (N)	Friability (%)	Ref
Modified	Tablets	FDM	10/50/90	4-ASA	PVA	385.7 (10% infill) 483.3 (50% infill) 484 (90% infill)	0	[90]
				5-ASA		333.3 (10% infill) 483.6 (50% infill) 483.4 (90% infill)		
	Hexagonal caplets	FDM/HME	60	Hydrochlorothiazide		86 (size A) 47 (size B) 20 (size C)	< 0.1	[91]
	Diamond caplets					134.24 (size A) 103.24 (size B) 113.35 (size C)	< 0.1	
	Tablets		20/100	Paracetamol	HPMCAS LG HPMCAS MG HPMCAS HG Methylparaben NF Magnesium stearate	high strength	0	[92]
			25/50/75	Ibuprofen	HPMCAS MG	94.3–460.7	0	[31]
			100	Captopril	HPC SL PEG 6000	411.3	0	[75]
		Direct extrusion	100	Theophylline	Eudragit^®^ RL GMS TEC	339.4	0.01	[93]
					Eudragit^®^ RL:RS (50:50) GMS TEC	340.4	0.02	
					Eudragit^®^ RS GMS TEC	342.3	0.02	
Controlled	Bilayer tablet	Extrusion	--	Guaifenesin	HPMC 2208/2910 PAA MCC SSG	69–118	< 1	[94]
	Tablets	SSE	--	Naftopidil	D-Mannitol PEG 4000 1–2% HPMC 2208 gel Crospovidone CL-F	20.7 [40% (1% HPMC 2208 gel)] 12 [50% (1% HPMC 2208 gel)] 43 [40% (2% HPMC 2208 gel)] 17.7 [50% (2% HPMC 2208 gel)]	--	[95]
					D-Mannitol PEG 4000 HPMC 2208 Crospovidone CL-F Carmellose calcium (ECG-505) Carmellose (NS-300)	16.7–43.7		
		FDM/HME	100	Acetaminophen	HPMC E5 (45.5%) EC N14 (19.5%) Crospovidone CL-F (5%)	242.2 (3DP) 52.9 (EXT) 176.5 (PM)	--	[96]
					HPMC E5 (45.5%) HPC EF (19.5%) Crospovidone CL-F (5%)	343.2 (3DP) 69.6 (EXT) 141.2 (PM)	--	
					HPMC E5 (45.5%) HPC LF (19.5%) Crospovidone CL-F (5%)	343.2 (3DP) 73.5 (EXT) 204.9 (PM)	--	
					EC N14 (50%) Methacrylic acid- methyl methacrylate copolymer (1:1) (15%) Crospovidone CL-F (5%)	343.2 (3DP) 66.7 (EXT) 133.4 (PM)	--	
		FDM	0/10 25/5090 100	Fluorescein	PVA	Mechanically strong enough to handle without damage	--	[32]
	Donut shape tablet		--	Hydrochlorothiazide	PVA Mannitol PLA	Unaffected up to 200 N	0	[97]
	Intra- gastric floating tablets	FDM/ HME	25/50 75	Pregabalin	HPMCAS HG PEG 400	very high mechanical strength, impossible to test with conventional hardness tester	0	[33]
Sustained	Ellisoid-shaped gastric floating tablets	FDM/ HME	15	Propranolol HCl	PVA Glycerol	>800 (A direction) 69 (B direction) 79.8 (C direction)	--	[98]
			25			>800 (A direction) 206 (B direction) 155.1 (C direction)	--	
	Gastrofloating tablets	FDM	0	Itraconazole	HPC-SL PVP	16.9 (F1) 60.3 (F2) 140 (F3) 181 (F4) 202 (F5)	--	[99]
		Extrusion	30	Dipyridamole (8.5%)	HPMC K4M (15%) HPMC E15 (15%) MCC PH101 (30%) Lactose (26.5%) PVP K30 (5%)	7.5–8.5	0.35	[100]
			50	Dipyridamole (7.25%)	HPMC K4M (15%) HPMC E15 (15%) MCC PH101 (30%) Lactose (27.75%) PVP K30 (5%)		0.27	
			70	Dipyridamole (6.5%)	HPMC K4M (15%) HPMC E15 (15%) MCC PH101 (30%) Lactose (28.5%) PVP K30 (5%)		0.21	
	Tablets (ring, solid geometry)	Extrusion	--	Paracetamol	PVP K25 NaCCS Starch	24.72 (ring) 88.42 (solid)	0.62 (ring) 0.59 (solid)	[72]
Sustained/biphasic	Mini tablets	FDM	100	Nifedipine	Hydrosupport filaments (> 96% polyvinyl alcohol and polyethylene glycol)	413.5 (filaments) 403.1 (tablets)	0(filaments) 0 (tablets)	[101]
		FDM/HME			PEG 4000 Magnesium strearate HPC EC	223.1 (filaments) 11.4 (tablets)	0 (filaments) 0.3 (tablets)	
Delayed/controlled	DuoCaplets	FDM/HME	100	Paracetamol Caffeine	PVA	High tablet strength	0	[102]
Delayed	Disk shaped tablets	Screen printing	--	Paracetamol	PVP K30 Starch Glycerol Sunflower oil HPMC K4M	102.5 (S) 138.5 (M) 201 (L)	0.01 (L)	[16]
	Donut shaped tablets					61.5 (S) 60 (M) 61 (L)	0.04 (L)	
	Cuboid shaped tablets					149 (S) 281 (M) ** 306.5 (L) **	0 (L)	
	Oval shaped tablets					80.5 (S) ** 99 (M) ** 114.5 (L) **	0.07 (L)	
	Grid shaped tablets					92.5 (S) ** 153.5 (M) ** 161 (L) **	0.03 (L)	

API: active pharmaceutical ingredient, EC: ethyl cellulose, Eudragit^®^ RS and RL: water-insoluble methacrylate polymers, FDM: fused deposition modeling, GMS: glyceryl monostearate, HME: hot melt extrusion, HPC: hydroxypropyl cellulose, HPMC: hydroxypropyl methylcellulose, HPMCAS: hydroxypropyl methylcellulose acetate succinate, MCC: microcrystalline cellulose, NaCCS: croscarmellose sodium, PAA: polyacrylic acid, PEG: polyethylene glycol, PLA: polylactic acid, PVA: polyvinyl alcohol, PVP: polyvinyl pyrrolidone, SSE: semi-solid extrusion, SSG: sodium starch glycolate, TEC: triethyl citrate, * Release rate as stated by the author(s). If the author(s) did not characterize the release rate, the following rule was applied: for drug release ≥ 75% within 30 min, the release profile was characterized as immediate. In every other case, the release profile was characterized as modified. ** The italics data entries indicate that the tablet was not broken in two parts, but rather bruised by the movable jaw of the hardness tester. Thus, the displayed braking force of the machine is rather an indicative reference rather than an absolute value, but it shows that these tablets are very robust to mechanical force.

In contrast to immediate-release products, modified-release dosage forms aim to optimize a therapeutic regimen by providing a slow and continuous delivery of the active ingredient over the entire dosing interval. Consequently, this leads to less frequent dosing and greater adherence to therapy [103]. According to the United States Pharmacopeia, “modified release” is a descriptive term for dosage forms with a drug release pattern that has been intentionally altered from the pattern observed in immediate release dosage forms of the same drug. There are two categories of modified release tablet formulations recognized by the Pharmacopeia: sustained release and extended release. Delayed release is a dosage form that has been intentionally modified to delay the release of the active ingredient for a period of time after initial administration. In contrast, extended-release tablets are formulated so that the active ingredient is available for a longer period of time after ingestion. Terms such as “extended release”, “controlled release”, or “delayed release” are often used as synonyms to describe such dosage forms [104]. However, in this literature review, the above terms are used strictly as indicated by the authors of the scientific articles included in Table 5.

Additive manufacturing offers a very wide range of possibilities to modify drug release by using different polymers, filling fractions, tablet geometries, etc. For example, Goyanes et al. manufactured modified drug release tablets: 4 ASA and 5 ASA with the FDM technique using PVA filaments. FDM proved to be effective for 5-ASA, but significant thermal degradation of the active 4-ASA (50%) occurred during printing, suggesting that this method may not be suitable for thermosensitive drugs. Moreover, tablets with three different filling percentages (10%, 50%, 90%) were printed and it was found that the tablets with higher filling percentage exhibited slower drug release as well as higher density and mechanical strength. All printed tablets showed very high hardness, between 330 and 390 N for the tablets with 10% filling and close to 485 N for those with higher filling percentage as well as zero friability [90]. A very similar strategy was followed by Nukala et al., who fabricated hydrochlorothiazide caplets using the FDM technique, where the drug was incorporated into PVA filaments by HME. However, the aim of their study was to investigate the effect of the printing pattern on the dissolution and physical properties of the final formulations, therefore two different patterns were used: hexagonal (HexCap) and diamond (DiaCap). The caplets were printed in three different sizes with dimensions (X, Y, Z) 15.0, 8.0, 3.99 mm (size A), 12.5, 6.44, 3.30 mm (size B) and 10.0, 5.30, 2.60 mm (size C) and the filling fraction was 60% for all formulations. The results of the friability test showed 0.1% for all caplets, but the hardness was significantly different for HexCap and DiaCap caplets, indicating that the internal structure of the caplets strongly influences their mechanical strength. DiaCap caplets exhibited higher hardness compared to HexCaps. Interestingly, the hardness of DiaCaps was almost the same regardless of the dimensions, but for HexCaps, the hardness decreased with decreasing caplet size. The printing pattern also had an effect on the dissolution profile, as HexCap appeared to dissolve faster than DiaCap. More specifically, HexCaps released about 80% of the drug in 45 min, while DiaCaps released only 50% of the drug in the same time. Finally, the drug release from the printed caplets was independent of the dimensions, as it did not differ between the different sizes of caplets (A, B, and C) [91].

Another scientific group also fabricated modified release paracetamol printlets from enteric polymers using the FDM/HME technique without the need of external enteric coating [92]. HPMCAS with different pH threshold were the main excipients used. More specifically, hypromellose acetate succinate (HPMCAS) is an enteric polymer that is a mixture of acetic acid and monosuccinic acid esters of hydroxypropylmethylcellulose. HPMCAS is marketed in three different grades depending on the ratio between acetyl and succinoyl groups—L, M and H—with pH limits of 5.5, 6.0, and 6.5, respectively [105]. According to the dissolution studies, the drug release from the printlets prepared with lower pH threshold polymers (HPMCAS LG, HPMCAS MG, HPMCAS HG) was faster. Moreover, formulations were printed with two different filling percentages (20%, 100%), the first resulting in faster drug release. Finally, it was reported that the mechanical properties of the printlets were satisfactory, with zero friability. However, the printlets exhibited a plastic-like aspect with high strength, which could not be quantified using a traditional tablet hardness tester.

Zhang et al. also experimented with HPMCAS to produce modified release ibuprofen tablets using FDM/HME technique [30]. For all formulations, 20 wt% ibuprofen was blended with 80 wt% HPMCAS MG polymers and the extruded filaments showed reasonable mechanical properties compared to commercial Tough PLA and Natural PVA filaments used as the reference. The researchers also conducted design of experiment (DoE) and recipe optimization studies using the Box–Behnken design to understand the correlation between structure and function of the printed tablets. More specifically, 15 batches of tablets were designed using different shell thicknesses, filling densities, and layer heights. For the tablet hardness test, two different orientations, 0°, and 45° were tested, where the probe movement aligned with the infill printing patterns or the diagonal direction of the crossed printing patterns, respectively. The results showed significant variation between two different orientations for most tablets. Moreover, no bending or splitting of the printed tablets was observed, mainly due to the strong adhesion force between adjacent layers. It was also found that the layer height may only affect the aesthetic details and not the tablet hardness, while the filling ratio and shell thickness play an important role in the mechanical properties of the final formulations. According to this study, high shell thickness and high filler density lead to stronger 3D printed tablets [30].

Hussain et al., in addition to the immediate release tablets discussed earlier, also prepared modified release tablets of captopril using the FDM/HME technique. The tablets were mainly composed of HPC-SL (binder) and PEG 6000 (plasticizer), which were carefully selected after a pre-formulation study (Table 3) to ensure that they imparted sufficient hardness to the final formulation. Indeed, the tablets showed a very high breaking strength (411.3 N), exceeding the upper limit of a common hardness tester as well as zero friability. According to the dissolution studies, only 50% of the drug was released in 2 h, indicating a modified release profile [75].

In addition, a very recent study describes the development of modified release theophylline tablets with direct extrusion 3D printing, eliminating the drying step after printing [93]. This was achieved by adding a fatty glyceride, glyceryl monostearate (GMS), and water-insoluble methacrylate polymers (Eudragit^®^ RL and RS). GMS acted as a plasticizer and lubricant at printing temperature, while it facilitated solidification at room temperature. Eudragit^®^ RL and RS, which have high and low water permeability, respectively, were used in different ratios (100% Eudragit^®^ RL, 100% Eudragit^®^ RS, Eudragit^®^ RL:RS 50:50) to test their influence on affecting the drug release profile. TEC was also used as a plasticizer due to its high miscibility with acrylic polymers. All 3D-printed tablets showed acceptable friability (1%) and very similar high breaking force values (339–342 N). This could be expected from polymer-rich 3D-printed tablets, which usually have very low friability and hardness values much higher than those of tablets produced by powder pressing. Finally, it was found that an increasing content of Eudragit^®^ RS resulted in a further delay in the release of theophylline from the matrix structure. This can be explained by the lower number of quaternary ammonium groups and consequently the lower hydrophilicity of Eudragit^®^ RS compared to Eudragit^®^ RL. Thus, by choosing the appropriate methacrylate polymer ratio, it is possible to tailor the drug release profile [93].

Finally, there is an interesting study on the development of a non-destructive method for the quality control of two separate active ingredients in a single 3D-printed tablet (polyprintlet) using NIR spectroscopy [106]. More specifically, they used selective laser sintering to produce cylindrical tablets with amlodipine and lisinopril as model active ingredients, polyethylene oxide (PEO) 100,000 as the thermoplastic polymer in the sintering process, and gold pigment to improve laser absorption and enable printability. PEO is a hydrophilic, linear, and uncrosslinked polymer available in a range of molecular weights from 100,000 to 8,000,000. It is commonly used in modified release formulations because it hydrates and swells upon contact with water, forming a hydrogel layer that delays drug release [107]. Three formulations were prepared with different drug concentration ranges (2–4% for amlodipine and 4–8% for lisinopril). The results of the hardness test showed that all formulations exceeded the maximum value that the device could measure as the tablets did not break, but were physically deformed (Table 6). In addition, the formulation with the highest drug content (4% amlodipine and 8% lisinopril) had the highest friability (0.93%). However, the friability of all formulations was less than 1%, making them suitable for handling and packaging. No dissolution studies were performed, so there are no data on the mechanism of drug release. Nevertheless, these excipients can potentially be used for the manufacture of modified release tablets and therefore their effects on the mechanical properties of the tablets may be useful in other future studies.

### 5.3. Controlled Release

Khaled et al. fabricated a two-layered guaifenesin tablet with immediate release (IR) and sustained release layers (SR) using an extrusion-based desktop 3D printer [94]. They sought to mimic the drug release profile of a commercial guaifenesin bilayer tablet by achieving an initial explosive release while maintaining therapeutic drug release levels over time. For the preparation of the IR layer, HPMC 2910 was used as the binder and microcrystalline cellulose and sodium starch glycolate as the disintegrant. For the SR layer, poly(acrylic acid) and four different percentages of HPMC 2208 (6%, 8%, 10%, 14% *w*/*w*) were used as the hydrophilic matrix. The printed tablets were subjected to hardness test and the results showed that the commercial tablets were more than two-fold harder than the printed ones as shown in the boxplot. It was also found that as the HPMC content increases, the tablet hardness also increases. As a result, the printed tablets containing 14% (*w*/*w*) HPMC 2208 exhibited the highest mechanical strength, although it was much lower than that of the commercial tablets. There was also a significant variation in friability between the printed and commercial tablets, mainly due to the low HPMC 2910 content in the IR layer, which resulted in poor bonding and consequently, high percentage weight loss. However, the friability of the printed tablets with 14% *w*/*w* HPMC 2208 was closest to the commercial tablets. Finally, all the printed tablets showed hardness within the accepted range of 69–118 N and reasonable friability (1%), indicating that they can be easily handled without losing their structural integrity [94].

Another scientific group produced controlled release naftopidil tablets with a 3D bio-printer using HPMC 2208 hydrogel (1% or 2%) as ink [95]. Mannitol, PEG 4000, and crospovidone CL-F were also included in the ink composition. They first prepared tablets with different percentages of HPMC gel without incorporating the drug (Table 7) and found that tablet hardness decreased with increasing percentage of HPMC gel. This is in contrast to a previous study [94] where increasing the percentage of HPMC resulted in an increase in mechanical strength. According to Tagami et al., this could be due to the different flow properties of the hydrogels in the different formulations. If higher pressure was applied during the extrusion process, the tablets produced could contain less water and thus have higher hardness.

Next, drug loaded tablets were produced that exhibited the same hardness tendency with increasing HPMC content; however, two formulations (30% [1% HPMC gel]) and (30% [2% HPMC gel]) could not be produced. This is mainly because the water soluble mannitol was replaced by the poorly water soluble naftopidil, which resulted in increased viscosity of the ink and hence difficulty in extrusion from the die. Of the four drug formulations tested, only the formulation (40% [2% HPMC gel]) resulted in tablets with sufficient hardness (43 N). Finally, three different types of disintegrants were used to investigate their influence on tablet properties: crospodidone CL-F (source type), NS-300 (wick type), and ECG-505 (bifunctional). Unexpectedly, it was found that the dissolution of the drug remained similar or was delayed when the amount of explosive incorporated was increased as opposed to conventional tablets prepared using a tableting machine. This may be attributed to the interaction of the disintegrants with the HPMC hydrogel, which contains a large amount of water and may alter the structure of the disintegrants, leading to loss of function. Finally, incorporation of crospovidone into the printer ink hardened the tablets, while incorporation of ECG-505 and NS -300 had little effect. These results indicate that the incorporation of crospovidone into the printer ink is effective in producing tablets using this technique [95].

In addition to extrusion-based 3D printing, FDM has also been used to fabricate controlled-release formulations. For example, Zhang et al. fabricated paracetamol tablets using the FDM/HME technique. Different grades and ratios of pharmaceutical polymers were tested to formulate filaments and tablets with the desired mechanical properties. PLA filaments without drug deposition were used as a reference standard to compare the differences between commercially available filaments and extruded filaments. Preliminary tests in which individual polymer formulations were prepared showed that HPMC filaments exhibited high breaking stress and high breaking distance. The HPMC filaments could be inserted into the printer, but printing was difficult due to the rough surface and high melt viscosity. The EC filaments had sufficient breaking stress, but were very brittle (small breaking distance) and were easily broken by the feed gear. The HPC LF and HPC EF filaments were too soft and flexible to be fed into the printer, while the methacrylic acid–methyl methacrylate copolymer (1:1) could not be extruded into filaments at high temperatures because it melted completely. These conclusions led to specific polymer combinations to produce filaments with a high breaking tension and a long breaking distance, allowing optimal printing (Table 5). Crospovidone type CL-F was also added to the formulations to smooth the surface of the filaments and reduce friction during the feed of the 3D process. In addition to the 3D printed tablets, physical mixtures of each formulation were compressed into tablets (PM tablets) and the extruded filaments were milled and compressed into tablets under the same conditions (EXT tablets). The results of the hardness test showed that the 3D-printed tablets had higher density and hardness than the directly pressed tablets. Moreover, some 3D-printed tablets exceeded the upper limit of the hardness tester (343 N). Finally, all 3D printed tablets showed good prolonged drug release rates due to their hardness and dense structure. On the other hand, faster drug release rates were observed for the directly compressed EXT and PM tablets [96].

FDM was also used by Goyanes et al., who fabricated controlled release fluorescein tablets to evaluate different printing parameters. PVA filaments were loaded with fluorescein by swelling the polymer in ethanolic drug solution. The final drug loading was low (0.29% *w*/*w*), but the use of other solvents could lead to higher encapsulation and/or greater diffusion into the polymer strands. In addition, tablets were printed with six different filling levels (0%, 10%, 25%, 50%, 90%, 100%). Tablets with 0% fill were hollow as only the shell was printed. Tablets with 10%, 50%, and 90% filling showed different internal patterns, which became denser with increasing filling degree, which in turn increased the mechanical strength. Finally, it was found that a higher filling percentage resulted in a prolonged release of the contained active ingredient. More specifically, the tablets with 10% filling showed complete release after 6 h, while 50% and 90% tablets released fluorescein for a longer period (77% and 70% drug release after 6 h, respectively). Complete dissolution of the drug took 15 h for 50% infill tablets and 20 h for 90% infill tablets [31].

Another study describes the fabrication of controlled release hydrochlorothiazide tablets by fused deposition modeling [97]. They developed a three-part donut-shaped (hollow cylindrical) solid dosage form with top and bottom layers consisting of water-insoluble, slowly biodegradable PLA caps and an intermediate layer of a water-soluble PVA/mannitol mixture loaded with hydrochlorothiazide. According to the dissolution studies, this hollow formulation showed zero order release kinetics up to 240 min. In contrast, in the case of the hydrochlorothiazide market product, most of the drug was released within 10 min. Finally, the 3D-printed tablets showed very good mechanical properties, with zero friability and a virtually unaffected hardness up to 200 N, which was the maximum applied force in the hardness tester.

### 5.4. Gastro Floating Tablets

In certain circumstances, prolonging the gastric residence time of a delivery system is desirable to achieve greater therapeutic benefit of the drug substance. For example, drugs that are absorbed in the proximal portion of the gastrointestinal tract, drugs that are less soluble in or degraded by alkaline pH, or drugs intended for local administration in the stomach may benefit from prolonged gastric retention [108]. Over the years, various approaches have been taken to fabricate gastroretentive dosage forms such as mucoadhesive, swelling, high-density, and floating systems [109].

**Figure 5:**
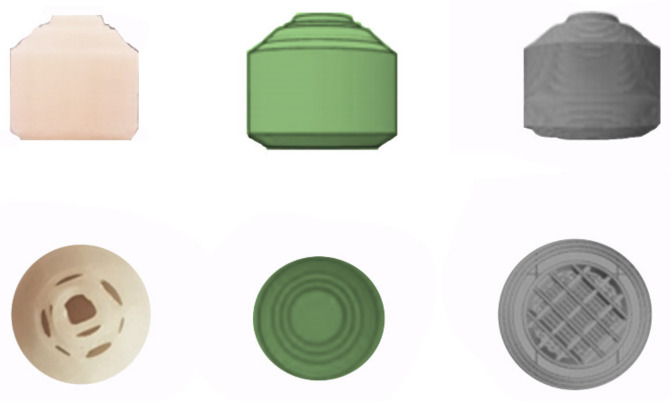
3D-printed pregabalin gastrofloating tablets. The internal structure of tablet is composed of a grid infill with void space filled with air so that the tablet has low density, which helps the buoyancy of the tablet in media [32].

The capabilities of 3D printing have been used to fabricate such systems, particularly gastrofluidic systems. For example, intra-gastric floating tablets with controlled release of pregabalin have been fabricated using the FDM technique [32]. Pregabalin has been shown to be absorbed primarily in the stomach and upper gastrointestinal tract, therefore, increasing gastric retention of the formulation reduces the frequency of administration. Various excipients were tested for the preparation of the drug-loaded filaments with HME, but only the filament prepared with 40% HPMCAS, 50% API, and 10% PEG 400 showed sufficient strength and flowability and was therefore suitable for extrusion and printing. Furthermore, the printed tablets showed very high mechanical strength, which could not be tested with a conventional hardness tester, while the friability was completely zero. In addition, three different filling percentages (25%, 50%, 75%) were tested to control the drug release. It was found that tablets with low fill percentages exhibited faster, but incomplete drug release compared to tablets with higher fill percentages. Finally, a unique geometry design was used in which one side of the tablet was closed, the other side was partially opened (Figure 5), and the fill was maintained at 25%. This design helped to achieve complete drug release while maintaining buoyancy for 24 h [32].

Chen et al. prepared propranolol hydrochloride gastrofloating tablets using the FDM/HME technique. PVA was the main excipient for filament production, while glycerol was also used as a plasticizer to lower the melting point of PVA. The tablets were printed with two different filling percentages (15 and 20%), which were carefully selected to ensure both floatability and hardness of the tablets. The rectilinear grid was used for the filling pattern of the 3D printed tablets to increase the mechanical strength [98]. The hardness test was performed in three directions and showed directional anisotropy between directions A and B and between directions A and C, which was assumed to be related to the layered structure of the tablets. More specifically, in direction A, both 3D-printed tablets exceeded the maximum detection range of 800 N. In direction B, the hardness of the tablets printed with 15% filling (E-15) and the tablets printed with 25% filling (E-25) was measured to be 69 N and 206 N, respectively, and in direction C, it was 79.8 N for E-15 and 155.1 N for E-25. Each layer of the tablet had a bottom layer for support when the hardness was measured in the A direction, so that the tablet could withstand a larger force and was difficult to break, while the layers of the tablet in the B or C direction slipped and cracked easily, so the hardness had a smaller value than in the A direction. No significant difference in hardness was found between directions B and C for both E-15 and E-25 [98]. This directional anisotropy was also reported by Zhang et al., who prepared modified release ibuprofen tablets as mentioned previously. During the hardness test, they found that the tablets exhibited higher strength when the force was applied at 0° than at 45° [30]. It was concluded that the hardness also depends on the direction of the probe with respect to the pressure pattern.

It was suggested that denser grids inside the tablet may provide more support, so that the tablet with higher filling ratio had greater hardness. Finally, the filling ratio also affected the floating behavior and drug release. E-15 tablets had a longer in vitro floating time, but faster drug release. It is referred that the tablet with a larger filling ratio contained more PVA and the diffusion of the drug and the erosion of excipients would take more time, suggesting that the larger filling ratio was responsible for the slowing of drug release [98].

Another scientific group produced itraconazole delayed release floating tablets using a FDM 3D printer [99]. HPC was used as the main excipient to provide the 3D printed tablets with a solid structure and a long floating time. PVP was also added to convert itraconazole from a crystalline state to amorphous state and improve its solubility. Five different formulations were prepared, all with a hollow structure (filling 0%) but different outer shell thickness. The results of the hardness test showed that as the thickness of the outer layer (side layer, top layer and bottom layer) increased, the tablet hardness also increased. High tablet hardness is less likely to collapse the tablet and allow fluids from the gastrointestinal tract to enter the hollow structure. It was also found that increased shell thickness resulted in delayed drug release and prolonged float time. This was confirmed by the fact that the tablets exhibited excellent mechanical strength (202 N), floated for a long period of time (540 min), and showed near zero drug release for 720 min at top and bottom shell thicknesses of 0.5 mm and side shell thickness of 1.5 mm [99].

In addition to the FDM process, extrusion-based 3D printing has also been used to fabricate gastroretentive delivery systems. Li et al. fabricated dipyridamole gastro-floating tablets using this technique [100]. Since dipyridamole is a water-insoluble and ethanol-soluble drug, the HPMC E15 hydro-alcoholic gel was prepared as a liquid binder. HPMC K4M was also used as a release inhibiting gel forming matrix, PVP K30 as the binder, lactose as the filler, and MCC PH 101 to improve the flowability of the paste and increase the deposit formability for the 3D printing process. The tablets were designed with three types of filling percentages (30, 50, 70%) and a lattice-like internal structure. It was found that lower filling percentage (30% and 50%) resulted in longer floating time due to lower density and higher air content. All formulations showed at least 8-h gastro-floating sustained release profile with hardly any floating lag time. In addition, all tablets exhibited acceptable friability (1%), although tablets with 30% fill were more fragile than tablets with 50% and 70% fill, as expected. Finally, all formulations possessed a hardness of 7.5–8.5 N. It is reported that a hardness greater than 6 N could potentially increase the floatation lag time or prevent flotation altogether, so conventional gastrofloating tablets trade hardness for shorter flotation lag time and greater buoyancy. However, these 3D-printed tablets barely achieved a floating delay time and prolonged buoyancy, while they were able to withstand a reasonable amount of rough handling without breaking or losing their structural integrity [100].

### 5.5. Sustained Release

In addition to paracetamol immediate release tablets with mesh geometry, Khaled et al. also produced ring and solid tablets from the exact same formulation using an extrusion-based 3D printer. PVP K25, NaCCS, and starch were used as excipients [72]. Surprisingly, the mesh tablets exhibited an immediate release profile, while the ring and solid tablets exhibited a delayed release profile, suggesting that geometry and surface area affect the release behavior of the drug, even for formulations containing disintegrants. In addition, the mechanical properties of the printed tablets were evaluated. The breaking force measurements were within the accepted range for the solid tablets (88.42 N), but did not reach the minimum satisfactory value for the ring tablets (24.72 N). In addition, there was a significant difference in tensile strength between the two geometries; the ring tablets exhibited an average tensile strength of 2.63 Mpa, while the solid tablets achieved a much higher value of 20.47 Mpa. Finally, as expected, solid tablets showed slightly lower friability (0.59%) than ring tablets (0.62%). However, both tablet geometries appeared to be quite robust and could tolerate a reasonable amount of rough handling [72].

### 5.6. Sustained-Biphasic Release

Ayyoubi et al. fabricated nifedipine mini-tablets by the FDM technique using modified commercial PVA filaments loaded by passive diffusion (PD) and ethyl cellulose-based filaments (EC) prepared by HME [101]. HME achieved very high drug loading (60%) compared to 3.7% of passive diffusion. Moreover, the mechanical properties of PD formulations and EC50 formulations (composed of 50% *w*/*w* nifedipine, 10% *w*/*w* EC, 34% *w*/*w* HPC, 5% PEG 4000, and 1% magnesium stearate) were compared. The friability test showed no weight loss for the mini-tablets and filaments prepared by the method PD, while no defects were visible in either the mini-tablets or the filaments at the end of the tests. On the other hand, a weight loss of 0.3% was observed in the EC50 mini tablets. According to the results of the hardness test, both the PD filaments and the mini-tablets were significantly firmer than the EC50 (Table 1). More specifically, the EC50 HME mini-tablets were 35 times more fragile than the PD mini-tablets, mainly due to the higher layer height during the printing process (0.12 mm for the PD and 0.23 mm for the EC50 formulations). The layer height is practically the thickness of each layer of the deposited material and can affect the hardness and structural integrity of the final formulation. The layer height was increased to ensure better flow of the molten polymer in the printer. However, the higher the layer height, the higher the risk of losing the resolution and altered surface during printing. Moreover, the density of the filament loaded with PD was 2–3 times higher than that of the HME, which is explained by the porous structure of the latter. Thus, the use of commercial filaments to produce the PD mini-tablets resulted in prints with better resolution and greater hardness. According to the dissolution studies, the PD mini tablets showed sustained release over 6 h, while the HME E50 mini tablets showed biphasic release with zero-order two phases. Finally, channelized E50 mini-tablets were designed with the aim of increasing the surface area exposed to the dissolution medium. Despite the increase in surface area, the channelized mini-tablets failed to improve the dissolution rate. This may be related to the formation of a gel-like layer around the mini-tablet, which limits dissolution and inhibits the effect of the increased surface area. It can be concluded that the composition of the formulation has a greater effect on drug release than the geometry [101], in contrast to the previous study by Khaled et al., who were able to modify the release properties only by changing the tablet geometry [72]. This could be explained by the fact that the drug content in the final formulation of the previous study [72] was much higher than the drug content in this study, so the effect of excipients on drug release in the first case is negligible compared to the geometry [101]

### 5.7. Delayed/Controlled Release

Goyanes et al. used the FDM technique to create a novel device for oral acetaminophen and caffeine. More specifically, it is a two-compartment device consisting of a caplet embedded in a larger caplet (DuoCaplet), and each compartment contains a different drug. Both drugs were incorporated into PVA filaments by HME. After dissolution testing, DuoCaplet showed a promising delayed/controlled release profile: the drug incorporated in the outer layer is released first and there is a delay time before the release of the drug contained in the core begins, depending on the properties of the outer layer. Finally, the mechanical properties of the device were excellent, with zero friability and incredibly high tablet strength, which is impossible to quantify with a conventional tablet hardness [102].

### 5.8. Delayed Release

Moldenhauer et al. used 3D screen printing to produce delayed-release paracetamol tablets on a large scale. The layout of the printing screen was designed in such a way that tablets with five different geometries (disk, donut, cuboid, oval, and grid), each in three different sizes (small, medium, large) could be produced during the same production process [15]. The results of the hardness test showed that all tablets had very good mechanical properties and high resistance to crushing. Moreover, tablets with non-circular base (cuboid, oval, lattice) were not broken into two pieces but crushed by the moving jaw of the hardness tester, proving that they are very robust to mechanical force. The donut tablets of all sizes had a constant breaking force of about 60 N, in contrast to the other tablet shapes where an increasing tendency to hardness was observed with increasing size (Table 5). Indicatively, the breaking force for the disks is reported to increase with size from about 100 N for the S size to about 200 N for the L size. Finally, all tablets exhibited extremely low friability (0.1%), from which it can be concluded that the physical tablet properties of 3D screen-printed tablets are by no means of lower quality than those of conventionally pressed tablets [15].

## 6. Computational Approaches in Pharmaceutical 3D Printing

### 6.1. General Considerations

Computational methods combined with 3D printing technology can open a new path for the design and construction of 3D tablets [110]. This method allows for the fabrication of structures with complex microstructures as well as good control and accuracy. Computational approaches are also valuable tools for predicting the structural properties and subsequent desired drug release, leading to a significant reduction in experimental iterations.

### 6.2. Printability, Drug Loading, and Drug Dissolution

Rheological characterizations are currently underrepresented in the 3D printing of formulations. In a study, viscosity data were used to develop a mathematical model that predicts the printability of fused deposition modeling 3D-printed tablets (printlets) [111]. Moreover, a prediction model has been developed to facilitate drug loading by passive diffusion on filaments that are used for FDM 3D-printing of oral dosage forms. Based on Hansen solubility parameters (HSP) and the concept of HSP distances (Ra) between drug, solvent, and filament, this pre-screening tool can estimate material miscibility and select the most appropriate combination that can provide a high drug loading [41]. Moreover, machine learning models (ML) have been built to predict the release behavior based on viscosity measurements. In contrast, the release profile was predicted using two components: partial least squares (PLS) analysis and rheological data. Only the viscosity measurements, according to the study, can be used for simultaneous high-throughput screening of printable formulations with the given release profile. By adjusting the porosity of the tablet, the drug release profile can be changed efficiently. The method correctly predicted the drug release rate for both single and multiple porosity tablets [112].

### 6.3. Design of 3D Structure

In this context, a computational approach to the fabrication of porous materials was designed and implemented [113]. The technique was used to create porous materials from polymer filaments such as polylactic acid and polyurethane. A commercially available dual nozzle melt deposition system was used to fabricate the geometries. It can be used with a variety of additive manufacturing processes, scales, and materials for a wide range of potential applications.

Another application of computational methods has been the use of material extrusion to develop porous 3D structures [114]. The volume preserving model for 3D printing has been proposed as a new computationally efficient method (VOLCO). The VOLCO model simulates material extrusion during fabrication and generates a 3D geometric representation of the expected microarchitecture. If previously printed 3D filaments interfere with the deposition of this new material, it is deposited into the nearest neighbor voxels based on a minimum distance criterion. With this technology, structures can be tested and optimized before production.

### 6.4. Artificial Intelligence Methods

Since the advent of artificial intelligence in recent years, many studies have attempted to improve the design and fabrication of scaffolds using neural networks. A study used an artificial neural network to investigate the simultaneous effects of layer thickness, lag time, and pressure direction on the porosity and compressive strength of the scaffold [115]. Other researchers have used a neural network method in addition to pressure settings to evaluate the structural properties of scaffolds to determine the appropriate mechanical behavior for tendon and ligament regeneration [116].

Using fused deposition modeling, artificial neural networks have also been used to develop a model to understand and predict diazepam release from printed tablets [117]. A computer-aided design application was used to create diazepam printed tablets with different shapes produced by fused deposition modeling. A self-assembling map and multilayer perception were used to model the effect of tablet surface area over volume ratio and printing parameters on drug release from 3D printed tablets. The estimated f2 factors for two tested formulations showed similarities between the experimentally observed and projected drug release profiles [117].

### 6.5. Limitations

Computer-aided methods are increasingly used in research for the design and fabrication of 3D structures. However, there are major limitations and difficulties that hinder effective simulation, modeling, and fabrication [118]. Since the microstructure of the scaffold is often on the micrometer or even nanometer scale, the various 3D printing methods lack the precision required to design structures of this size.

## 7. Discussion

After a comprehensive study of the current literature, some conclusions were drawn that could contribute to the fabrication of 3D printed oral dosage forms with desired mechanical properties. It was also noticed that during the first years of research on 3D-printed oral dosage forms, FDM or extrusion based 3D-printing were mainly applied for the fabrication of tablets, usually with a single API, basic geometry (round or oval), and average mechanical properties [31,94]. However, very soon, numerous excipients were screened and tested and new methods were developed, leading to more specialized formulations such as gastrofloating and chewable tablets, polypills, and complex-shaped tablets with improved mechanical and release properties.

Binder jetting produces highly porous structures, often with low mechanical strength, and is therefore best suited for the production of immediate release, fast dissolving, or orodispersible dosage forms. The binder is important in controlling tablet breaking force in 3D printing, as opposed to the compressive forces that control tablet breaking force in conventional tableting presses. Fillers with high water solubility, humectants with high water content, and binders with high viscosity in solution can increase tablet hardness and binding force.

As for pediatric formulations, since the vast majority are chewable or orally dispersed dosage forms, BJ and/or extrusion-based 3D printing are usually preferred, among other techniques. In BJ-printed pediatric formulations, it was found that the use of PVP K30 as a binder at a certain percentage (0.05% *w*/*w*) resulted in a significant decrease in friability, while hardness remained unchanged. Additionally, the addition of glycerol to the ink in proportions within 4% (*w*/*w*) was able to significantly improve the formability of the tablets, however, a higher glycerol content (6–8% *w*/*w*) resulted in very high friability values (9%). Moreover, in extrusion printed gum formulations, increasing the amount of gelatin led to an increase in mechanical strength. Incorporating HPMC 2208 into the formulation increased viscosity but had no effect on strength or decreased it slightly, while adding the active ingredient had no effect on hardness. Finally, in chocolate-based formulations, corn syrup used to facilitate drug loading resulted in a decrease in hardness, which was desirable since these dosage forms are intended for pediatric patients.

SLS is another technique that offers the advantage of controllable porosity and is therefore most commonly used for the preparation of orodispersible formulations. Copovidone VA 64 has been found to be an important excipient for ODTs due to its good printability and fast disintegration properties. It produces tablets with adequate mechanical properties and also helps printlets to retain their structure even at low levels (~15%). Moreover, the laser scanning speed in SLS has a statistically significant effect on the mechanical properties. The optimal laser speed for ODTs or immediate release tablets is 200–300 mm/s. Formulations prepared at these laser scanning speeds are lighter and have higher porosity, which is due to the fact that increasing the speed reduces the sintering of the powder particles together and leaves more voids between each particle. Apart from speed, microcrystalline cellulose was also shown to have a statistically significant effect on the hardness of SLS-printed tablets. It increased the mechanical strength due to its high binding capacity and also showed a synergistic binding effect in combination with copovidone VA 64.

For immediate release tablets produced by thermal extrusion 3D printing, an increase in PEG 4000 resulted in an increase in hardness while keeping the friability consistently low. However, there is another study that suggests the use of other binders since PEG 4000 has poor bonding and consequently unacceptable hardness and friability values.

Regarding modified release dosage forms, FDM is mainly used as it produces tablets with complex geometries, excellent hardness (> 300 N), and low friability (< 1%). Apart from the auxiliary materials, temperature also affects the mechanical properties. A high temperature (above the Tg of the polymers) during the printing process allows molecular diffusion and thus strong interfacial adhesion between the adjacent layers of the molten material. However, too high a temperature could create air pockets between the layers and lead to porosity, reducing the mechanical strength of the final formulation. In conclusion, it is emphasized that FDM is the technique of choice only for thermoresistant agents.

Apart from excipients, several printing parameters have a great impact on the physical properties of printed tablets. The percentage of filling is a valuable tool to modify not only the release profile, but also the mechanical strength, depending on the needs of the patient. A high fill percentage results in high breaking strength and slow drug release. The printing pattern is also worth considering when high tablet hardness is desired. For example, the diamond pattern has a higher hardness than the hexagonal pattern. In addition, shell thickness is a printing parameter that plays a statistically significant role in mechanical properties. A high shell thickness generally results in mechanically stronger 3D printed tablets. Layer height, on the other hand, may only affect esthetic details and not mechanical properties.

For 3D-printed gastrofloating tablets, it was found that a lower shell thickness leads to a longer floating time due to the lower density and higher air content. In addition, as the shell thickness increases, the intra-gastric flotation time increases. Finally, FDM and extrusion-based 3D printing are mainly used for the fabrication of controlled release tablets. In SSE, incorporation of crospovidone type CL-F into the printer ink is recommended as it increases tablet hardness. HMPC 2208 is another excipient commonly used to modify release properties, although it provides conflicting observations as far as mechanical properties are concerned. When increasing amounts of HPMC 2208 were used in the SR layer of a controlled release bilayer printlet, an increase in hardness was observed. On the other hand, when HPMC 2208 hydrogel was used for the preparation of extrusion-based controlled release tablets, the tablet hardness decreased with increasing amount of HPMC gel.

According to a recent systematic review that included 131 articles from 1999 to 2020, FDM was the most widely applied 3D-printing technique for the fabrication of solid oral dosage forms [119]. As far as characterization tests are concerned, the hardness test was mostly performed in the SLS included articles, with a percentage equal to 83%, whereas in FDM, only half of the articles (51%) reported an evaluation of the mechanical strength. Friability tests were performed in an even smaller number of publications (FDM: 24%, SLS: 17%) (Table 8). Therefore, thus far, there are limited data at our disposal to draw safe conclusions about the mechanical properties of solid oral dosage forms. Taking all these into consideration, for the quality of 3D-printed oral formulations, it is important in future works to carry out this test, to evaluate whether the 3DP drug delivery systems are suitable or not for the intended use, and if they meet the pharmacopeia specifications.

After careful evaluation of the recent limited literature, it could be suggested that FDM is the technique that leads to a harder tablet. This is supported by the fact that most of the tablets with the highest breaking force values (> 400 N) that have been included in this review were fabricated with FDM [32,75,90,98,101]. Furthermore, among all the excipients used, PVA filaments seem to be the optimum choice for the production of oral dosage forms with zero friability and excellent hardness [90,101] that can even exceed 800 N [98].

Considering all these factors, it can be concluded that the mechanical properties of 3D printed oral dosage forms are a complex but important topic that needs to be further investigated with extensive pre-formulation studies. In this way, optimization of printing processes will be possible so that 3D printing can reach the peak of its pharmaceutical potential in the near future.

Finally, in silico methods, used in conjunction with 3D printing technology, could pave the way for new techniques to design and build 3D tablets. Computational techniques are useful for predicting structural features and consequent intended drug release, resulting in a large reduction in experimental iterations. However, there are some limitations today, largely due to the lack of precision in the various 3D printing processes. In the future, computational studies can investigate more accurate techniques to model scaffolds to avoid geometric discrepancies with manufactured parts. In addition, more accurate mechanobiological models should be investigated to simulate various tissue applications and simulate the properties and behavior of scaffolds under multi-physics conditions. Further studies are needed to increase the adaptability, precision, and resolution of different 3D printing techniques, which will support the fabrication of computationally designed complex biomimetic scaffolds.

## 8. Conclusions

Three-dimensional printing is a new manufacturing technology that is gaining attraction in the pharmaceutical industry to move away from mass production and toward personalized pharmacotherapy. In the study, critical aspects of 3D printing are discussed such as hardness, tensile strength, friability, and infill content, which are important parameters that affect the mechanical strength of the printed tablet. The advantages and disadvantages of the technologies used to construct 3D printed tablets were also analyzed such as binder jetting, fused deposition modeling, semi-solid extrusion, selective laser sintering, and stereolithography. A literature search revealed a number of applications of 3D printing in pediatric formulations and orodispersible tablets as well as modified release, biphasic release, gastro floating tablets, and sustained release tablets. Finally, computational methods and artificial intelligence methods in combination with 3D printing technologies are valuable tools for predicting structural properties and subsequent drug release, leading to a significant reduction in experimental effort.

## Figures and Tables

**Table 1:** Composition of 3D-printed solid immediate release oral dosage forms linked with breaking force (N) and friability (%).

Dosage Form	Technique	API	Excipients	Breaking Force (N)	Friability (%)	Ref
Cartoon pediatric preparations	BJ /color jet	Levetiracetam	MCC PH101 Mannitol Sucralose PVP K30 Glycerin Polysorbate 20 Isopropanol	~ 60 (0% PVP K30) ~ 56 (0.03% PVP K30) ~ 56 (0.05% PVP K30) ~ 55 (0.1% PVP K30) ~ 58 (0.2% PVP K30) ~ 68 (0.5% PVP K30)	~10.5 (0% PVP K30) ~ 8 (0.03% PVP K30) ~ 6 (0.05% PVP K30) ~ 5.5 (0.1% PVP K30) ~ 6 (0.2% PVP K30) ~ 7 (0.5% PVP K30)	[64]
				~ 56 (0% Glycerin) ~ 51 (0.5% Glycerin) ~ 48 (1% Glycerin) ~ 44 (2% Glycerin) ~ 49 (4% Glycerin) ~ 59 (6% Glycerin) ~ 42 (8% Glycerin)	~ 6.5 (0% Glycerin) ~ 6 (0.5% Glycerin) ~ 6.5 (1% Glycerin) ~ 6.5 (2% Glycerin) ~ 5 (4% Glycerin) ~ 9 (6% Glycerin) ~ 8 (8% Glycerin)	
			MCC PH101 Mannitol Sucralose PVP K30 (0.05%) Glycerin (4%) Polysorbate 20 Isopropanol	13 (Hollow with internal support) 61 (Hollow) 59 (Lattice)	Hollow structure with internal support failed the friability test. Hollow structure and lattice structure remained intact	
Gummy formulations	SSE	Lamotrigine	Gelatin HPMC 2208 Reduced starch syrup	~ 4 (0 mg HPMC 2208) ~ 4 (100 mg HPMC 2208) ~ 3.8 (200 mg HPMC 2208) ~ 3.7 (300 mg HPMC 2208)	--	[65]
				~ 1.5 (0.5 g Gelatin) ~ 2 (0.75 g Gelatin) ~ 3.8 (1 g Gelatin) ~ 8 (1.5 g Gelatin)		
				~ 3.8 (0 mg API) ~ 3.8 (10 mg API) ~ 3.7 (20 mg API)		
Chewable dosage forms (various shapes)	Extrusion	Paracetamol Ibuprofen	Bitter chocolate Corn (glucose) syrup	4.99–14.6	--	[66]
Orodispersable tablets	SSE	Carbamazepine	HPβCD (72.1%) HPMC F4M (1.4%) NaCCS (2.5%)	25	2.2	[67]
Orodispersable tablets	SLS	Ondansetron	Food grade β-Cyclodextrin (MW 1135 g/mol) Copovidone VA 64 (25%) Mannitol (50%) Gold pigment	14.7 (Copovidone VA 64 (25%) Mannitol (50%))	--	[68]
				18.5 Copovidone VA 64 (15%) Mannitol (60%)		
Orally disintegrating tablets	SLS	Paracetamol	HPMC E5 (92%) Gold pigment (3%)	144.3 (100 mm/s) 52.7 (200 mm/s) 15.7 (300 mm/s)	--	[69]
			Copovidone VA 64 (92%) Gold pigment (3%)	171.2 (100 mm/s) 27.3 (200 mm/s) 13.7 (300 mm/s)		
				14.5 (no pattern) 13.9 (Braille letter A) 14.3 (Braille letter Q)	--	[70]
Tablets	SSE	Carbamazepine	HPβCD (72.1%) HPMC F4M (3.9%)	35	1.8	[67]
	Extrusion	Paracetamol	PVP K25 NaH_2_PO_4_ and Na_2_HPO_4_ NaCCS	78.1	0.54	[71]
Tablets (mesh geometry)	Extrusion	Paracetamol	PVP K25 NaCCS Starch	24.67	0.65 (mesh)	[72]
Tablets	TE	Puerarin	PEG 4000/API (3:1) PEG 4000/API (4:1) PEG 4000/API (5:1) PEG 4000/API (6:1)	79 93 118 138	0.23 0.21 0.23 0.16	[73]
	SLS	Clindamycin palmitate HCl	Copovidone VA 64 MCC LMH Blue pigment Iron (III) Oxide	7.3–18.3	--	[74]
	FDM/HME	Captopril	HPC SL PEG 6000 Sodium starch glycolate	406.4	0	[75]
			HPC SL PEG 6000 NaCCS	409.9		
Gaplets	FDM/HME	Theophylline	HPC-SSL Triacetin	227.5 (0.0 mm) 135 (0.2 mm) 77.5 (0.4 mm) 53.6 (0.6 mm) 37.9 (0.8 mm) 25.2 (1.0 mm) 16.7 (1.2 mm)	0	[76]
Tablets	BJ	Amitriptyline HCl	LMH PVP K30 Di-Calcium phosphate anhydrate	35–49	< 0.87	[77]
Solid lipid tablets	SSE	Fenofibrate	Glycerol/Glyceryl monolinoleate (mixed long-chain glycerides) Glycerol Tricaprylate/Tricaprate (medium-chain triglycerides) Glyceryl Caprylate/Caprate (mixed long-chain glycerides) Soybean oil Polyethoxylated castor oil Polysorbate 85 Methylcellulose NaCCS	Appropriate mechanical properties for easy handling A tablet hardness test could not be performed	--	[78]

API: active pharmaceutical ingredient, BJ: binder jetting, FDM: fused deposition modeling, HME: hot melt extrusion, HPC: hydroxypropyl cellulose, HPMC: hydroxypropyl methylcellulose, HPβCD: hydroxypropyl-β-cyclodextrin, LMH: lactose monohydrate, MCC: microcrystalline cellulose, NaCCS: croscarmellose sodium, PEG: polyethylene glycol, PVP: polyvinyl pyrrolidone, SLS: selective laser sintering, SSE: semi-solid extrusion, TE: thermal extrusion.

**Table 6:** The mechanical properties of SLS printed tablets with different concentrations of two separate active pharmaceutical ingredients [106].

API	Excipients	Breaking Force (N)
Amlodipine (2%) Lisinopril (4%)	PEO 100.000 (91%) gold pigment (3%)	>483.7
Amlodipine (3%) Lisinopril (6%)	PEO 100.000 (88%) gold pigment (3%)	>484
Amlodipine (4%) Lisinopril (8%)	PEO 100.000 (85%) gold pigment (3%)	>483.7

**Table 7:** Hardness of extrusion printed tablets without the drug, prepared by incorporating different amounts of HPMC hydrogel into the printer ink [95].

Excipients	Breaking Force (N)
30% (1% HPMC 2208 gel) 55% Mannitol 10% PEG 4000 5% Crospovidone CL-F	33.7
40% (1% HPMC 2208 gel) 45% Mannitol 10% PEG 4000 5% Crospovidone CL-F	23.7
50% (1% HPMC 2208 gel) 35% Mannitol 10% PEG 4000 5% Crospovidone CL-F	18.7
30% (2% HPMC 2208 gel) 55% Mannitol 10% PEG 4000 5% Crospovidone CL-F	34.7
40% (2% HPMC 2208 gel) 45% Mannitol 10% PEG 4000 5% Crospovidone CL-F	30
50% (2% HPMC 2208 gel) 35% Mannitol 10% PEG 4000 5% Crospovidone CL-F	20.3

**Table 8:** Schematic summaries of the two characterization tests (hardness; friability) performed with the four 3DP techniques (FDM; SLS, SLA; inkjet 3DP) and quantification in how many articles were evaluated [119].

Technique	Hardness	Friability
FDM	51%	24%
SLS	83%	17%
SLA	37.5%	0%
Inkjet 3DP	14%	21%
